# Phylogeography and adaptation genetics of stickleback from the Haida Gwaii archipelago revealed using genome-wide single nucleotide polymorphism genotyping

**DOI:** 10.1111/mec.12215

**Published:** 2013-03-04

**Authors:** Bruce E Deagle, Felicity C Jones, Devin M Absher, David M Kingsley, Thomas E Reimchen

**Affiliations:** * Department of Biology, University of VictoriaVictoria, British Colombia, Canada, V8W 3N5; † Department of Developmental Biology, Stanford UniversityStanford, CA, 94305-5329, USA; ‡ HudsonAlpha Institute for BiotechnologyHuntsville, AL, 35806, USA; § Howard Hughes Medical Institute, Stanford UniversityStanford, CA, 94305-5329, USA

**Keywords:** *Gasterosteus*, population structure, single nucleotide polymorphism

## Abstract

Threespine stickleback populations are model systems for studying adaptive evolution and the underlying genetics. In lakes on the Haida Gwaii archipelago (off western Canada), stickleback have undergone a remarkable local radiation and show phenotypic diversity matching that seen throughout the species distribution. To provide a historical context for this radiation, we surveyed genetic variation at >1000 single nucleotide polymorphism (SNP) loci in stickleback from over 100 populations. SNPs included markers evenly distributed throughout genome and candidate SNPs tagging adaptive genomic regions. Based on evenly distributed SNPs, the phylogeographic pattern differs substantially from the disjunct pattern previously observed between two highly divergent mtDNA lineages. The SNP tree instead shows extensive within watershed population clustering and different watersheds separated by short branches deep in the tree. These data are consistent with separate colonizations of most watersheds, despite underlying genetic connections between some independent drainages. This supports previous suppositions that morphological diversity observed between watersheds has been shaped independently, with populations exhibiting complete loss of lateral plates and giant size each occurring in several distinct clades. Throughout the archipelago, we see repeated selection of SNPs tagging candidate freshwater adaptive variants at several genomic regions differentiated between marine–freshwater populations on a global scale (e.g. *EDA*, *Na/K ATPase*). In estuarine sites, both marine and freshwater allelic variants were commonly detected. We also found typically marine alleles present in a few freshwater lakes, especially those with completely plated morphology. These results provide a general model for postglacial colonization of freshwater habitat by sticklebacks and illustrate the tremendous potential of genome-wide SNP data sets hold for resolving patterns and processes underlying recent adaptive divergences.

## Introduction

Observations of species’ phenotypic adaptations to local environments have inspired and informed generations of evolutionary biologists. Parallel adaptive changes under replicated ecological conditions, such as the repeated evolution of ecomorphs in *Anolis* lizards (Losos 2009) or the parallel diversification of cichlid fish (Kocher 2004), have been particularly valuable for understanding evolutionary processes. Many of these adaptive divergences have been extensively studied at the genetic level, with most of the focus on collecting phylogenetic information to clarify their historical context (e.g. Losos *et al*. 1998; Allender *et al*. 2003). Phylogenetic data can reveal how many independent times various phenotypic transitions occurred, the directionality of change, and provide a timeline for the adaptations (Avise 2006). At the intra-specific level, phylogeographic surveys have typically employed quickly evolving mitochondrial DNA (mtDNA) or microsatellite markers. However, with recent advances in DNA sequencing and high-throughput genotyping, genome-wide data sets are beginning to be collected from wild populations allowing more robust reconstruction of the structure and demographic histories of populations (e.g. Willing *et al*. 2010). By contrasting the levels of divergence across the genome, these population analyses also allow areas of the genome under selection to be identified (Beaumont & Balding 2004). With this ability to identify outlier loci, and through the use of classical genetic approaches (such as QTL mapping), the genetic basis of the traits involved in adaptive divergences is increasingly being revealed (Elmer & Meyer 2011). Phylogeographic surveys incorporating adaptive genetic markers in addition to putatively neutral markers will allow insight into whether parallel adaptive genetic changes are occurring across many populations and will allow new understanding of environment–phenotype–genotype interactions.

Radiation in form, physiology and behaviour of threespine stickleback (*Gasterosteus aculeatus*) has been the focus of a diverse range of studies examining evolutionary processes (reviewed in Wootton 1976; Bell & Foster 1994). This small fish is widely distributed in marine and coastal fresh waters of the northern hemisphere (Wootton 1976). Most of the diversification of stickleback occurred after morphologically conservative anadromous stickleback colonized freshwater habitats that were created when ice sheets of the most recent glaciation receded (<15000 ybp). One of the most striking examples of morphological differentiation in vertebrates occurs in threespine stickleback from lakes on the Haida Gwaii archipelago, off western Canada. Among islands and within watersheds, these fish display a remarkable diversity in adult body size, longevity, defence morphology, trophic structures and nuptial pigmentation which equal or exceed that found throughout the entire species distribution (Moodie & Reimchen 1976b; Reimchen *et al*. 1985; Reimchen 1994; Spoljaric & Reimchen 2007). Cases of extensive armour loss (i.e. populations lacking all lateral plates or completely missing the pelvic girdle) and major differentiation in adult body size are particularly well-studied (Moodie & Reimchen 1976b; Reimchen 1983, 1994; Reimchen *et al*. 1985; T. E. Reimchen, C. A. Bergstrom and P. Nosil unpublished data; Gambling & Reimchen 2012). Natural selection drives much of the differentiation seen in these insular populations (Moodie 1972; Reimchen 1980, 1983, 1994, 2000; Reimchen & Nosil 2002). However, as the populations in adjacent watersheds, or over small geographic areas, may not represent independent colonization events, it is not clear how many separate times various divergent stickleback phenotypes have arisen on Haida Gwaii.

Phylogeographic data based on mtDNA have been collected for Haida Gwaii stickleback revealing two highly divergent mitochondrial lineages estimated to have separated over a million years ago (O’Reilly *et al*. 1993; Orti *et al*. 1994; Deagle *et al*. 1996). The distribution of one lineage, only in freshwater populations near postulated ice-free refugia, was initially interpreted as evidence of extended existence of this lineage in the region (O’Reilly *et al*. 1993). A subsequent global study showed the divergent mtDNA ‘refugia’ lineage was ubiquitous in Western Pacific samples collected around Japan (Japan Sea lineage) and formed a clade distinct from European and most North American populations (ENA lineage) (Orti *et al*. 1994). The presence of this ancient mtDNA polymorphism and otherwise low level of sequence divergence between Haida Gwaii populations has obscured the relationships among populations.

Over the last 10 years, stickleback research has been brought to the forefront of ecological genomics through the development of a suite of resources, including genome sequences of 21 individuals from diverse marine and freshwater populations and a high-quality threespine stickleback reference genome (Jones *et al*. 2012b). This has allowed the genetic basis for several adaptive traits to be determined (Colosimo *et al*. 2005; Miller *et al*. 2007; Chan *et al*. 2010). One of the best studied is the sticklebacks’ lateral plate armour. Marine fish are characterized by a row of ∼35 bony plates running down each side of the body (completely plated), this contrasts with freshwater populations which generally retain <10 plates (low plated). Intermediate (partially plated) also occur. The difference in plate morphs has a relatively simple genetic basis with more than 70% of variation in plate number controlled by the Ectodysplasin gene (*EDA*) (Colosimo *et al*. 2004). Selection for lower lateral plate number in freshwater populations has been attributed to many potential mechanisms (reviewed in Bell 2001) and is almost universally due to shifts between two allelic forms of *EDA* (Colosimo *et al*. 2005). Freshwater populations of Haida Gwaii are mostly low plated (generally six or seven plates), but variation encompasses the complete range with lake populations having means from 0 to 30 lateral plates (T. E. Reimchen, C. A. Bergstrom and P. Nosil 2013).

Beyond the allelic shift seen at the *EDA* locus during colonization of freshwater, a large number of other parallel genetic changes differentiate marine and freshwater stickleback (Hohenlohe *et al*. 2010; Jones *et al*. 2012a,b). These parallel genome-wide changes have been identified using genome scan approaches in limited range of marine and freshwater populations and therefore their generality across populations is not clear. Recent work on Haida Gwaii stickleback has also identified some genomic regions under divergent selection between adjoining stream and lake populations (Deagle *et al*. 2012). Somewhat paradoxically some of these stream-lake outlier loci are also highly differentiated in studies considering marine–freshwater divergence. This indicates that certain marine adaptive variants are retained in at least some freshwater populations. By documenting the distribution of these genetic variants, it may be possible to identify commonalities between the marine and freshwater populations which share adaptive genomic regions and narrow the search for candidate genes.

Here, we use a stickleback genome-wide genotyping array with >1000 single nucleotide polymorphism (SNP) markers (Jones *et al*. 2012a) to document genetic variation in a comprehensive geographic survey of Haida Gwaii stickleback. Most markers on the array were chosen to be evenly distributed across the stickleback genome. Data from these SNPs provide a multilocus view of relationships between populations producing a historical framework in which to examine this remarkable morphological radiation. Within a phylogeographic context, it will be possible to determine whether cases of extreme morphological variation (e.g. lake populations with body gigantism or major loss of body armour) are due to a convergence or common ancestry. These data will also be useful to evaluate potential extended residency of freshwater stickleback in glacial refugia that existed between Haida Gwaii and the mainland (see Reimchen & Byun 2005; for discussion). This geographic region has a complex Pleistocene history and is at the centre of debate surrounding the human coastal migration theory (Josenhans *et al*. 1997). The phylogeographic picture that emerges will also provide a general model for patterns of postglacial colonization of freshwater habitat by sticklebacks on a local scale and should provide insight into drivers of genetic diversity within freshwater.

In addition to evenly spaced SNPs, candidate SNPs linked to potentially adaptive genomic regions were also genotyped. These SNPs primarily tag genomic regions identified as being differentiated between marine and freshwater populations (e.g. EDA, Na/K ATPase Jones *et al*. 2012a) and their inclusion allow us to map their distribution throughout a large number of populations on the archipelago. We address some specific questions regarding these adaptive variants: do all low plated populations share the characteristic freshwater *EDA* haplotype found in most other surveyed populations? Do the completely plated freshwater populations retain the typical marine *EDA* haplotype and/or do they retain other marine-like genomic regions? More generally we compare marine–freshwater divergence within our data set with previous genome-wide analyses (Hohenlohe *et al*. 2010; Jones *et al*. 2012b). We also further document the distribution of haplotypes identified as outliers between stream-lake populations from Haida Gwaii (Deagle *et al*. 2012).

## Materials and methods

### Stickleback samples

**Fig 1:**
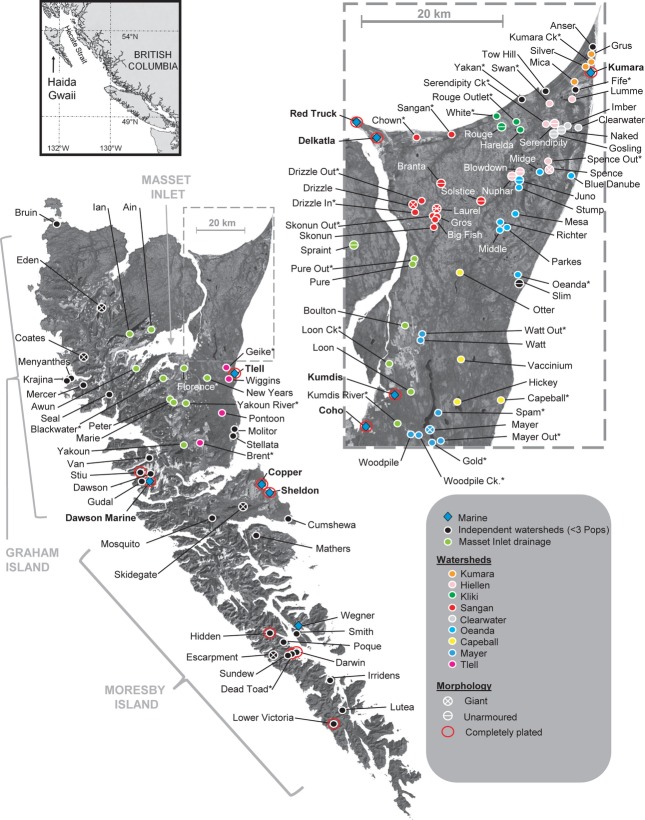
Haida Gwaii localities where threespine stickleback were collected. Populations which drain into Masset Inlet and those from watersheds with greater than two collection localities are colour coded to illustrate connections. Symbols identify marine/estuarine sampling sites and morphologically distinct populations (completely plated, unarmoured and giant). An asterisk beside the population name indicates a stream population.

From the Haida Gwaii archipelago, a total of 462 stickleback from 115 localities representing 54 watersheds were genotyped (Fig. 1; Table S1, Supporting information). Specimens (*n* = 5) from the mid-Pacific Ocean (45°31′N, 179°24′W) were also genotyped as archetypal Pacific marine fish. We maximized the number of populations sampled to obtain a broad survey comparable in scope to previous morphological analyses (T. E. Reimchen, C. A. Bergstrom and P. Nosil 2013). Due to the large number of populations considered, only two individuals were genotyped for most locations; however, in 15 populations ≥10, fish were analysed [including eight populations from a previous study on adjacent stream-lake pairs (Deagle *et al*. 2012)]. The low number of individuals genotyped per site meant that some common population genetic analyses based on population allele frequency estimates were inappropriate. Sample localities covered three major physiographic regions (lowland, plateau and mountain; see Sutherland-Brown & Yorath 1989) and were classified as lake (*n* = 77), stream (*n* = 28) or marine/estuarine (*n* = 10). Morphological variation between sampled populations encompassed the extremes seen within the species. Here, we have highlighted (i) ‘unarmoured’ populations with extensive loss of bony lateral plates [12 populations with a mean of less than one lateral plate on left side of fish (T. E. Reimchen, C. A. Bergstrom and P. Nosil 2013), Fig. 1] and (ii) ‘giant’ populations with the largest recorded body lengths [eight populations identified in (Gambling & Reimchen 2012), Fig. 1]. Collections were made using minnow traps primarily in spring/summer of 2009 and 2010 (samples stored in 95% ethanol). Additional samples were from collections made in 1993 (see Deagle *et al*. 1996). For one location (Harelda), stickleback from both 1993 (*n* = 6) and 2009 (*n* = 6) were genotyped to confirm samples were comparable.

### SNP genotyping

Genomic DNA was extracted from muscle tissue and 1536 biallelic SNP loci genotyped using Illumina’s BeadArray Technology and GoldenGate assay (Illumina, San Diego, USA) following Jones *et al*. (2012a). SNPs were originally identified in two marine and three freshwater populations distant from Haida Gwaii (>800 km) and are distributed across all 21 linkage groups, mtDNA and unassembled scaffolds (see Jones *et al*. 2012a). The SNPs can be classified into three groups: (i) SNPs chosen to be evenly distributed across the genome based on local recombination rate; (ii) SNPs chosen to tag unoriented or unassembled genomic regions; and (iii) candidate SNPs targeting regions differentiated between marine and freshwater populations identified in previous studies (Colosimo *et al*. 2005; Jones *et al*. 2012a,b) or potentially linked to traits of interest based on published studies on homologous traits in other diverse organisms. The SNPs genotyped here are the same as those in Deagle *et al*. (2012); these include those SNPs with good genotyping signals from Jones *et al*. (2012a) along with additional candidate SNPs. genomestudio software (v 2010.2; Illumina, San Diego, USA) was used to visualize intensity signals. Genotypes were initially called automatically, then position of all intensity clusters were visually inspected and adjusted manually. SNPs with poorly separated loose clusters or exhibiting low signals were excluded from further analysis. SNPs missing >10% of genotypes calls and any individuals with >5% missing data were excluded. Repeatability of calls was >99% for individual DNA samples genotyped multiple times. The final data set included 1170 SNPs (773 evenly distributed, 117 genome assembly and 280 candidate SNPs; Table S2, Supporting information) from 467 stickleback (462 Haida Gwaii, five mid-Pacific Ocean).

### Population heterozygosity

Population heterozygosity was calculated as mean of individual observed multilocus heterozygosities based on all evenly distributed SNPs (excluding sex-linked loci; *n* = 760, hereafter referred to as the evenly spaced SNP data subset). Given the large number of loci, individual heterozygosity estimates are precise (randomly dividing the SNP loci in half and calculating individual heterozygosity for both sets of loci yields a median coefficient of variation (CV) of 5.0%). Variance between individuals within a population was also small (in populations where at least 4 individuals were genotyped the mean CV, within populations, was 8.5%). This suggests estimates of relative population heterozygosities are robust even with SNP data from only a few individuals.

We examined population heterozygosity as a function of habitat type (lake, stream, marine). For lake populations, we also assessed correlations between heterozygosity and three physical parameters (distance from ocean via outlet, elevation and lake area) by fitting linear models. The relative importance of the independent variables (and confidence intervals calculated based on 1000 bootstraps) was determined using the r package relaimpo (Gromping 2006).

### Tree-base analysis

Individual-based distance trees were produced with two arbitrarily selected stickleback from each locality and using data from the evenly spaced SNP data subset (760 loci). These trees were constructed in mega version 5 (Tamura *et al*. 2011) using the neighbour-joining (NJ) algorithm based on a pairwise uncorrected P distance matrix (equivalent to allele sharing distance Gao & Starmer 2007) calculated from an artificial nucleotide sequence created by concatenating each individual’s diploid SNP data (missing data coded as N). Substitution of different individual stickleback from the same populations (where more than two individuals were genotyped) had only minor impact on tree branching patterns at nodes with low bootstrap support.

### Principal component analysis

Principal component analysis (PCA) is an effective approach for dimension reduction of multivariate data sets and has been widely adopted in the analysis of SNP data sets as an unsupervised method to identify underlying structure (Patterson *et al*. 2006). For PCA of archipelago-wide genetic structure, we used data from the evenly spaced SNP data subset and carried out separate analyses using two arbitrarily selected individual stickleback per population and using population allele frequencies based on all individuals. PCA requires a data set without missing values so we filled in missing entries (0.7% of individual data, 0.1% of population data) by randomly sampling data for that locus across all localities (separate re-samplings had very minor impact on PCA clustering). We used the function *prcomp* in r statistical software (v 2.9.0) to perform the PCA (R 2009). A *k*-means clustering algorithm, also implemented in r, was used to assign individuals/populations to clusters based on the SNP data (10 independent runs were used to confirm stability of clustering).

### MtDNA analysis

The Haida Gwaii data set included 10 mtDNA SNPs, including two SNPs known to differentiate the Japan Sea and ENA lineages (cytochrome b gene position 564 and 690 from Orti *et al*. 1994). We used these SNPs to further map the distribution of the Japan Sea lineage on Haida Gwaii. We also genotyped a larger sample of individuals from two lakes (Serendipity and Harelda) known to contain both mtDNA lineages (O’Reilly *et al*. 1993) to investigate whether intra-population mtDNA clustering was reflected in nuclear DNA markers, or whether any evidence could be found for selection on the joint mitochondrial-nuclear genotype. Fish from these two lakes were typed with a mtDNA lineage diagnostic restriction enzyme test (see Deagle *et al*. 1996) prior to genome-wide SNP genotyping ensuring approximately equal numbers of fish from each lineage (Serendipity *n* = 17: eight ENA, nine Japan Sea and Harelda *n* = 16: eight ENA, eight Japan Sea).

### Adaptive genetic variation

Inclusion of SNPs that are linked to alternate allelic forms of various adaptive loci allows us to map the geographic distribution of these alleles in Haida Gwaii populations. Our broad survey design and resultant limited sample sizes within populations precludes detection of local adaptive variation (i.e. only occurring in one or a few populations). Instead, we focused on allelic variants tagged by multiple SNPs on adaptive haplotypes documented across several populations in previous studies. These include SNPs tagging *EDA* (chr4—12.8 Mb) and *Na/K ATPase* (chr1—21.7 Mb: candidate gene for salinity tolerance differences), both loci are highly differentiated between marine and freshwater stickleback in several populations (Hohenlohe *et al*. 2010; Jones *et al*. 2012a,b). Allelic forms of these regions were each assessed at 6 tightly linked SNPs (*EDA*, chrIV: 12811933, 12814920, 12815024, 12815271, 12816360, 12831803 and *Na/K ATPase*, chrI: 21662413, 21672254, 21683350, 21689292, 21694776, 21701627). To examine the association between allelic forms of *EDA* and lateral plate phenotype, we scored all stickleback for lateral plate number (left side). We also looked for potentially novel genomic regions that are highly differentiated between marine and freshwater localities in our data set. To do this, we calculated the difference in allele frequency between a pool of all freshwater populations (*n* = 104) vs. a pool of all the marine/estuarine populations (*n* = 10) and compared the most divergent SNPs (>50% difference) to previously identified outlier regions (Hohenlohe *et al*. 2010; Jones *et al*. 2012b). We also carried out PCA of stickleback from all populations using just these divergent, habitat-associated SNPs to identify freshwater populations containing marine-associated alleles, or marine populations containing freshwater-associated alleles.

Finally, we consider SNPs identified by Deagle *et al*. (2012) as being highly differentiated between multiple Haida Gwaii stream-lake pairs of stickleback. As the same SNP genotyping array was used in both studies, all outliers could be geographically mapped based on the current data set. However, most of these are single SNPs representing large genomic regions and therefore are not ideal cross-population markers for the adaptive variants (i.e. they can become unlinked due to recombination or allelic variation). Here we consider two outlier regions (chr4: 19.8 Mb and chr19: 14.8 Mb Deagle *et al*. 2012) identified in stream-lake analysis and each defined here by three SNPs (chr4:19881291, 19881370 and 19881515 and chr19:14796728, 14798132, 14799088). Both these regions are also outliers in marine–freshwater comparisons (Hohenlohe *et al*. 2010; Jones *et al*. 2012b).

## Results

### Heterozygosity

**Fig 2:**
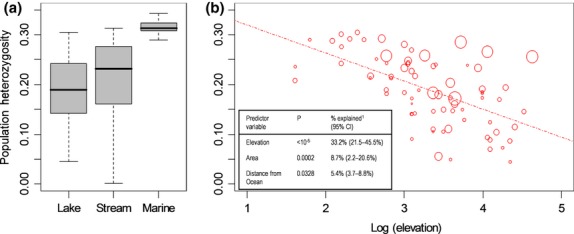
Population level heterozygosity of Haida Gwaii localities for evenly spaced single nucleotide polymorphisms. (a) Boxplots (median, range, upper/lower quartiles) showing heterozygosity in populations collected in different habitats. (b) Heterozygosity of lake populations plotted against their elevations; size of plotting symbol is proportional to lake area (fourth root). Inset shows a summary of a multiple regression model with three significant biophysical predictor variables for heterozygosity of lake populations. Overall *R*^2^ was 0.47, and percent of variation in heterozygosity explained by each variable independently is shown [calculated using a relative importance measure averaged over predictor orderings (Gromping 2006)], confidence intervals based on 1000 bootstraps.

Heterozygosity of Haida Gwaii populations for evenly spaced SNPs ranged from 0.002 to 0.343 (mean = 0.206 ± 0.078 SD) and was higher in marine localities compared to streams and lakes (Fig. 2a). The lowest heterozygosity was found in small ponds and headwater creeks; in one creek (Blackwater), the two fish were homozygous for the same allele in 758 of 760 loci. In lake populations, heterozygosity was correlated with three independent variables considered (log values of elevation, lake area and distance from ocean) (Fig. 2b). In a full multiple regression model, there were no significant interaction terms, with interaction terms removed all variables were significant (overall *R*^2^: 0.47). Percentage of variation in heterozygosity explained by each variable model (averaged over orderings) was as follows: lake elevation 33.2% (95% CI = 21.5–45.5%); lake area 8.7% (95% CI = 2.2–20.6%); distance from ocean 5.4% (95% CI = 3.7–8.8%).

### Tree-based analysis of population structure

**Fig 3:**
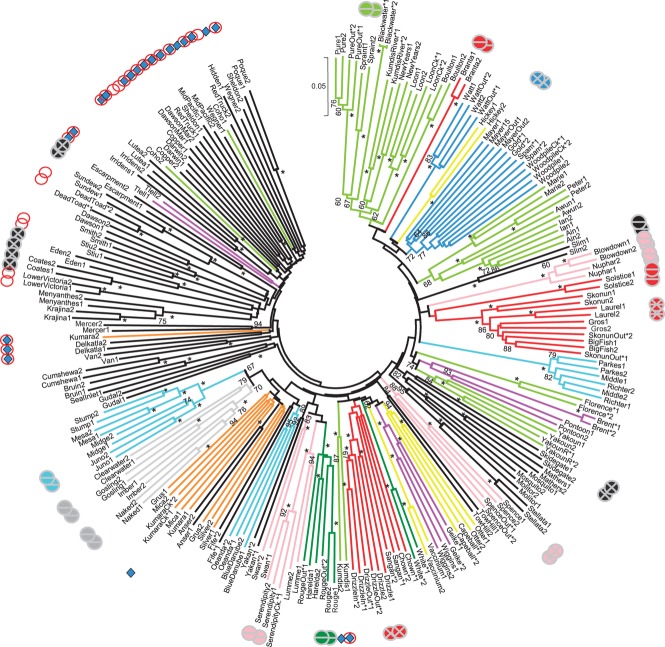
Neighbour-joining distance tree constructed with single nucleotide polymorphism (SNP) data from two stickleback from each of 115 Haida Gwaii localities (*n* = 227 individuals; three populations with single fish). Colours and symbols follow scheme in Fig 1. Tree constructed based on a pairwise uncorrected P distance matrix calculated using evenly spaced SNPs (*n* = 760); missing data were removed in a pairwise manner. Bootstrap values >50% are shown next to branches and values >96% are marked with an asterisk.

A distance-based tree constructed using two fish per collection site reveals several levels of genetic structuring in Haida Gwaii populations (Fig. 3). Genetic distances between individuals accounts for most separation within the tree, although there is considerable variation (i.e. some population harbour very low levels of genetic diversity—see heterozygosity section above). Individuals from the same population are almost universally grouped together at terminal nodes. The few cases where fish collected at the same locality do not cluster together occur at marine/estuarine sites, or when individuals from adjacent populations are interspersed (Fig. 3).

The next group of well-supported clusters primarily joins fish collected from common watersheds (Fig 3). For example, in the Sangan watershed, which contains a great deal of morphological diversity (see Reimchen *et al*. 1985), fish from Skonun Lake are grouped together with, along with adjoining streams and several nearby ponds (99% bootstrap support; Fig. 3; Detailed map in Fig. S1, Supporting information). There are several exceptions to the watershed-driven structuring. First, there are many cases where populations from within the same watershed are separated. For example, again within the Sangan watershed, fish from Drizzle lake, two isolated lakes and stickleback collected near the river mouth fall in separate or weakly supported clusters (Fig. 3; Fig. S1, Supporting information). Second, there are examples in which adjacent freshwater watersheds are joined in the tree. This is most prevalent in populations draining into a large saltwater inlet (Masset Inlet) on Graham Island (Fig. 3; Fig. S1, Supporting information).

The basal region of the tree is characterized by a large number of short branches that are poorly supported by bootstrap values. Despite being poorly supported, these branches deep within the tree still tend to group populations by geographic region. When a condensed tree is generated (50% bootstrap support cut-off value), the basal region of the tree collapses into an unresolved node with 80 independent branches (Fig. S2, Supporting information). Many of these branches contain only fish from one population (most often these are sole representatives for the watershed or marine/estuarine populations).

The 12 populations containing predominantly unarmoured stickleback are distributed across eight genetic clusters which each branch independently from the basal node of the tree (based on condensed tree). This is consistent with armour loss occurring independently in separate watersheds. These populations include unarmoured stickleback in two groups of headwater lakes in adjacent watersheds that are geographically close (<750 m apart) but genetically distant (Juno vs. Blowdown/Nuphar and Serendipity vs. Gosling/Naked; Detailed map in Fig. S1, Supporting information). Populations of giant stickleback show a similar pattern of independence in different watersheds, with the eight giant populations coming from seven distinct genetic clusters (based on condensed tree).

### PCA of population structure

**Fig 4:**
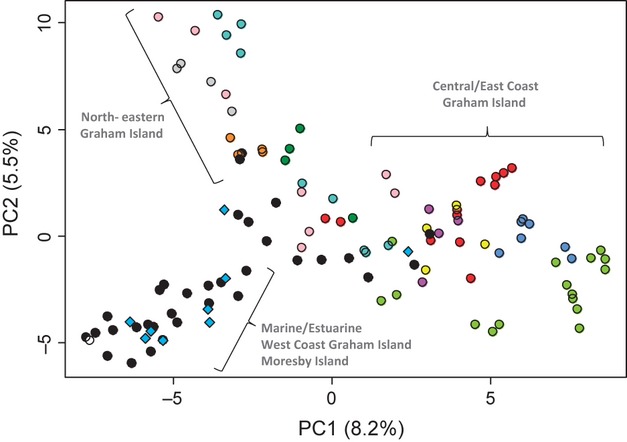
Principal component analysis reveals clustering of geographic regions based on population single nucleotide polymorphism (SNP) allele frequency data (evenly spaced SNPs; *n* = 760). Each point represents a sampling location, colours follow scheme in Fig. 1 with the mid-Pacific sample labelled white.

Principal component analysis partitioned genetic variation into three broadly congruent clusters regardless of whether individual genotypes or population level allele frequencies are considered (population level data presented here). Based on population allele frequencies, the first two PCs account for 8.2% and 5.5% of the variation respectively (Fig. 4; see Fig. S3 for population labels, Supporting information). With membership defined using k-means clustering (*k* = 3), the first cluster contains marine localities as well as freshwater populations from the entire western and southern regions of the archipelago. The second cluster is limited to localities on the north-east tip of Graham Island, including watersheds that flow both north and east into the ocean. The final cluster consists of a large number of Graham Island populations distributed from the Sangan watershed in the north through the central area and to the east coast (Fig. 4). Further PCs, and *k*-means clustering with higher values of k, tend to cluster small groups of populations from a single watershed. For example, PC3 (and *k*-means clustering *k* = 4) separates out three closely linked lakes in the headwaters of the Oeanda River (Parkes, Middle, Richter).

To investigate the number of SNPs that are driving separation of the three PCA clusters, we considered small groups of SNPs categorized according to their informativeness (relative weightings on eigenvector); by examining these SNPs, in turn, we evaluated how quickly the ability to approximate the observed clustering declined (see Fig. S3, Supporting information). PC1 and PC2 from the overall data set were highly correlated with their top ten SNPs indicating that the overall clustering observed can be obtained with 20 SNPs. However, it is not only these SNPs driving the clustering. Reasonable approximations could be obtained (*r*^2^ > 0.5) for any of the 10 SNP data subsets created from the 200 top-weighted SNPs on either of the first two principal components. This analysis suggests that a broad range of SNPs rather than a few selected SNPs are driving the observed PCA clustering.

### Comparison with previous mtDNA studies of population structure

Analysis of mtDNA SNPs identified 39 fish from 12 populations containing the Japan Sea mtDNA (Table S1, Supporting information). These included 10 populations where the lineage had previously been identified (O’Reilly *et al*. 1993; Deagle *et al*. 1996) and two west coast populations not included in prior studies (Menyanthes and Stiu). The distribution of the Japan Sea mtDNA did not correspond to overall clustering of the SNP data (i.e. this mtDNA lineage was distributed throughout the tree and in different PCA clusters). In the two mixed mtDNA populations, from which larger numbers of samples were genotyped (Serendipity and Harelda), fish did not cluster according to mtDNA lineage in NJ trees constructed based on evenly spaced nuclear SNPs (Fig. S4, Supporting information). No strong associations were found between any nuclear SNP and mtDNA lineage, so we have no evidence of co-adapted mitochondrial-nuclear genes.

### Geography of adaptive genomic regions

**Fig 5:**
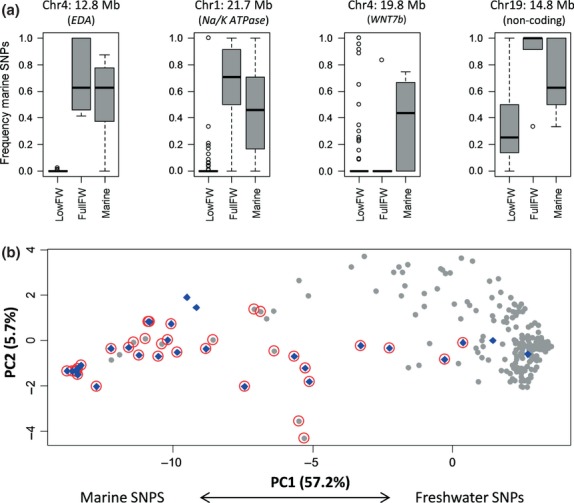
Population distribution of single nucleotide polymorphisms (SNPs) linked to alternate allelic forms of candidate adaptive loci (a) Boxplots showing frequency of four candidate loci in low-plated freshwater, completely plated freshwater and marine/estuarine populations. All four genomic regions have previously been shown to be divergent between marine and freshwater locations in global comparisons. Chr4:19.8 and Chr19:14.8 Mb were also previously identified as outliers between some Haida Gwaii parapatric stream-lake populations. (b) Principal component analysis plot showing positioning of individual stickleback based on subset of SNPs most divergent in allele frequency between marine and freshwater localities (*n* = 78; excluding SNPs linked to *EDA*). Stickleback from marine/estuarine collection sites are shown as diamonds, completely plated individuals are circled. See Fig. S5 (Supporting information) for list of SNPs and plot with populations labelled.

Changes in lateral plate phenotype, which generally occur following colonization of freshwater habitats, are almost universally attributed to two allelic forms of *EDA* (alleles: C = complete, L = low). All low plated fish we genotyped were homozygous for the L allele (as assessed at six tightly linked SNPs; Fig. 5a). All fish with complete or partial lateral plate phenotypes had at least one C allele, and this includes stickleback from 10 marine/estuarine localities with fish exhibiting a range of plate phenotypes. It also includes completely and partially plated fish from several freshwater lakes (Fig 5a). Of the four lake populations containing completely or partially plated fish, two (Darwin and Hidden) could potentially have had recent gene flow with the marine environment (heterozygosity = 0.291 and 0.208 respectively). Two other lake populations (Stiu and Lower Victoria) are isolated from the ocean by high-gradient streams and have correspondingly lower heterozygosity (0.151 and 0.056 respectively). Despite very overall low heterozygosity, Lower Victoria contains both the C and L allelic forms of the *EDA* gene.

The global marine–freshwater outlier genomic region near the *Na/K ATPase* gene (defined by six SNPs in the current analysis) was also highly divergent between these habitats in our data set (Fig 5a). The marine/estuarine populations contained many fish heterozygous for the alternate forms (consistent with variable plate morphology and the *EDA* locus pattern) and the freshwater haplotype was generally fixed in lakes and streams (Fig 5a). Exceptions in which marine SNPs are retained in freshwater primarily occur in completely plated freshwater populations (Fig 5a).

To examine general patterns of SNP divergences between marine and freshwater on the archipelago, we identified SNPs that diverged most in frequency between these habitats (86 SNPs from 28 genomic regions; for details see Fig. S5, Supporting information). Most of these divergent SNPs were in genomic regions characterized as outliers in previously marine–freshwater comparisons (23 of 28 regions were also outliers in either Hohenlohe *et al*. 2010; Jones *et al*. 2012b). A PCA based on these divergent SNPs (excluding SNPs linked to *EDA* to reduce the direct influence of plate morphology) produces a gradient of marine-like to freshwater-like fish along PC1 (Fig. 5b). The extremes of PC1 (explaining 57% of the variance) are represented by strong negative scores for the mid-Pacific samples and marine waters of Haida Gwaii (Dawson Marine) and strong positive loadings for most freshwater individuals (Fig 5b). Several marine/estuarine collected individuals clustered with freshwater fish, indicating they were freshwater residents. Other fish from these sites were intermediate, suggesting varying levels of admixture. Freshwater localities containing completely plated fish generally cluster closer to marine fish, indicating that these populations tended to retain a suite of marine-like alleles across the genome in addition to the SNPs at *EDA* and *Na/K ATPase* (for details see Fig. S5, Supporting information). However, this retained association with *EDA* C alleles is not universal. One notable example is Poque lake, a lake population that is low plated (both fish homozygous for *EDA* L), but otherwise contained many alleles usually found in marine stickleback. The chr11 5.7 Mb haplotype, a previously identified inversion differing in orientation between marine and freshwater ecotypes (Jones *et al*. 2012b), is almost invariant in freshwater, with Poque lake and most completely plated freshwater populations (e.g. Stiu and Hidden) matching the FW genotypes (Fig. S5, Supporting information).

The two previously identified stream-lake outlier regions (chr4: 19.8 Mb and ch19: 4.8 Mb, Deagle *et al*. 2012) that are also outliers in marine–freshwater comparisons (Hohenlohe *et al*. 2010; Jones *et al*. 2012b) were not strongly differentiated between stream and lake habitats in the archipelago-wide data set. The chr4: 19.8 Mb ‘lake haplotype’ (defined by 3 SNPs) was generally at a low frequency in freshwater populations; however, it was prevalent in the marine/estuarine samples (Fig. 5a). Some lakes where this haplotype was common contained giant stickleback (e.g. Laurel, Coates, Awun) a trait shared with lakes in which the outliers were originally described, although not all giant populations shared this haplotype. The ch19: 4.8 Mb shows a similar pattern (i.e. the lake haplotype is common in marine/estuarine sites and less common in freshwater), but the lake haplotype is present at an intermediate frequency in many freshwater populations (Fig. 5a). The retention of these marine-like haplotypes in relatively few lakes explains how these loci can be outliers in different studies comparing stream-lake and marine–freshwater populations.

## Discussion

Our survey of genetic variation in Haida Gwaii stickleback using a genome-wide SNP array helps refine 40 years of ecological and evolutionary research carried out on these morphologically diverse fish populations. The overall phylogeographic pattern observed in freshwater populations differs substantially from previous mtDNA analysis. Rather than the disjunct pattern observed between two highly divergent mtDNA lineages (O’Reilly *et al*. 1993; Deagle *et al*. 1996), the genome-wide population tree shows extensive within watershed population clustering and different watersheds are separated by numerous short branches deep in the tree. Despite some level of underlying connections between watersheds, the data are consistent with separate colonization of most watersheds. This supports previous suppositions that morphological diversity observed between watersheds is shaped independently and that the remarkable morphological divergences observed within some watersheds occurs despite overall genetic similarities. Our results also provide a geographic view of adaptive genetic differences at a regional scale. Almost all genomic regions that were strongly divergent between marine and freshwater populations on Haida Gwaii match those identified as divergent across this ecological gradient elsewhere; however, in a small number of lakes (predominantly those containing completely plated fish), many marine-like SNPs are retained.

### Genetic diversity, population history and genetic structuring

The low level of genetic variation observed in freshwater stickleback populations compared to marine fish is consistent with expectations due to increased genetic drift in smaller populations and potential for founder events during colonization (Bell 1976) and mirror previous results (Withler & McPhail 1985; Taylor & McPhail 1999; Makinen *et al*. 2006; Jones *et al*. 2012a). There was considerable variation in heterozygosity between lake populations allowing us to examine factors driving loss of genetic variability in freshwater. Heterozygosity was inversely correlated with lake elevation, and to a lesser extent distance from ocean. This indicates a role for bottlenecks during colonization; however, these data could also result from ongoing genetic enhancement of accessible freshwater populations via a low level of gene flow with marine/estuarine stickleback (e.g. Keller *et al*. 2001). The higher explanatory power of elevation compared to distance from ocean probably reflects increased metapopulation connectance in low-gradient streams due to the presence of stream resident stickleback and the fact that high-gradient streams represent major barriers to stickleback movement (Caldera & Bolnick 2008). Our finding that lake size was positively correlated with heterozygosity suggests genetic drift is also important in shaping current genetic diversity (assuming lake size is correlated with population size). The complete range of lake size is present at higher elevations, and high heterozygosity is retained in large high elevation lakes. Overall our data indicate that reduced heterozygosity in freshwater stickleback results from a range of processes including founder effects, genetic drift and ongoing gene flow.

There has been much interest in the phylogeographic structure of Haida Gwaii stickleback due to their extensive morphological diversification and potential for long-term persistence of some population in glacial refugia (Moodie & Reimchen 1976a; O’Reilly *et al*. 1993). Initial analyses of mtDNA variation were obscured by the presence of two ancient mtDNA lineages that originated in the two distinctive genetic groups of stickleback in the Pacific Basin (Japan Sea vs. remainder of the Pacific) (O’Reilly *et al*. 1993; Orti *et al*. 1994). These distinctive stickleback are considered separate species where they come into contact, but hybridization has led to apparently unidirectional introgression of mtDNA from the Japan Sea lineage, ultimately resulting in Japan Sea mtDNA in some Haida Gwaii populations (Orti *et al*. 1994; Yamada *et al*. 2001; Kitano *et al*. 2007). To examine whether mtDNA history is reflected in nuclear SNPs, or whether evolutionary dynamics of mtDNA are being driven by epistatic interactions with nuclear genes (see Dowling *et al*. 2008), we looked for associations between nuclear SNPs and mtDNA in populations containing both mtDNA lineages. No clear associations were identified, and the data set as a whole indicates that mitochondrial and nuclear genome have distinct evolutionary paths. Despite past reliance on mtDNA, it is apparent that this single genetic marker can provide a misleading view population history and our current analysis of hundreds of loci represented a much clearer view of genetic structuring.

There are no highly distinct freshwater genetic clusters in the SNP data to indicate that stickleback relic populations persisted in refugia near Haida Gwaii during the last glacial advance. These data do not reject the possibility; we simply see no clear evidence suggesting genetic continuity of a refugial lineage. This is not surprising given the dynamic geological processes occurring over the last 12 000 years. Any freshwater refugia that did exist would probably have been below present day sea level between Haida Gwaii and the mainland (Josenhans *et al*. 1995; Barrie & Conway 1999). While the continental ice sheet melted sea level rose to 12-m higher than present day, meaning many current Haida Gwaii freshwater lakes would have been inundated by marine waters (Josenhans *et al*. 1997). During colonization of newly formed habitat, there would have been numerous opportunities for gene flow between marine and freshwater stickleback, and perhaps between divergent freshwater populations (McKinnon *et al*. 2004), which could have blurred the genetic signal of a hypothetical refugial lineage.

The dominant features of the Haida Gwaii stickleback SNP-based tree are as follows: almost universal clustering of individuals from the same population, clustering of populations from within the same watershed and many branches connecting at a shallow central node (star phylogeny). Rather than a traditional phylogenetic tree where distance measures sequence divergence over time, our data set represents shuffling of standing genetic variation over time via population genetic processes. This does blur some relationships, for example, estuarine fish tend to follow the pattern of marine fish, branching off independently from the basal clade rather than clustering with their watershed, presumably due to ongoing gene flow. Small pond populations with low heterozygosity are also often only weakly linked to other populations within the watershed, likely due to genetic drift. Apparent associations between low heterozygosity populations also need to be interpreted with care as these populations may tend to fix the same common ancestral alleles and may also maintain shared variation at loci subject balancing selection. At first consideration, the placement of marine/estuarine populations into a common, albeit weakly supported, cluster along with several freshwater populations could be taken as evidence for very limited number of origins of freshwater stickleback on Haida Gwaii. However, clustering of marine stickleback is expected to some degree because contemporary marine stickleback are likely to be more similar to each other than to ancestral marine stickleback, and due to directional selection which differentiates some genomic regions between these habitats. The key point is that genetic distances between many freshwater populations are similar to distances between marine and freshwater environments. So, while we may never know how many independent origins of freshwater stickleback happened, the ancestral genes appear to have been almost completely shuffled between colonisations of freshwater watersheds (see also Berner *et al*. 2009).

While the pattern of branching indicates most freshwater watersheds can be treated as independent, it is clear that there are some well-supported genetic linkages between populations currently separated by marine waters. This is most apparent in populations surrounding Masset Inlet, a large saltwater inlet in central Graham Island. Two broad, nonmutually exclusive, scenarios could explain these connections: ongoing gene flow or a single freshwater origin across neighbouring watersheds. It seems unlikely that there is ongoing gene flow between all grouped Masset Inlet populations because some are currently inaccessible, highly morphologically distinct and have low heterozygosity (e.g. Pure and Spraint lakes). A single freshwater origin and dispersal in this region is feasible. When sea levels were lower during and immediately following the last glacial maximum (Josenhans *et al*. 1997; Barrie & Conway 1999), currently independent rivers would have coalesced in a large postglacial river flowing along the channel of Masset Inlet. Ancestral stickleback could have colonized this river, spread to tributaries, and then been isolated in the newly formed headwater lakes when Masset Inlet was flooded by rising sea levels *c*. 9000 ybp (Josenhans *et al*. 1995). However, more recent gene flow between freshwater stickleback populations from adjoining watersheds cannot be discounted, indeed some Masset Inlet populations (e.g. Kumdis River and Loon Outlet) are currently separated by only a few kilometres of coastline and show higher genetic affinities than more distant watersheds.

Principal component analysis gives a slightly different perspective on genetic structuring of Haida Gwaii stickleback by highlighting similarities within three clusters that are separated by short basal branches in the SNP tree. Three overlapping clusters identified by PC1 and PC2 correspond to broad geographically cohesive areas, further principal components cluster only small numbers of populations. Of the three broad clusters, one includes all marine stickleback as well as freshwater populations extending over a large part of the archipelago, from Moresby Island to the west coast of Graham Island. Another cluster includes populations from central Graham Island (including watersheds draining east and north) and the final cluster consists of populations from the north-east lowland area of Graham Island. These groups are not being driven by a small number of shared SNPs, with several hundred SNPs showing this underlying structure. It seems unlikely that this connectivity between watersheds across the archipelago is maintained solely by recent gene flow via marine waters, because freshwater clusters do not group watersheds draining into a common marine basin and because marine fish cluster together regardless of collection location. We have considered several possible alternative explanations. One scenario is that colonization of freshwater occurred in successive waves, resulting in two distinctive freshwater lineages and a third group of more recently derived populations similar to marine stickleback. This is speculative as the present pattern would only have developed if historical drainages connected some watersheds currently flowing east and north on Graham Island. Furthermore, we have no a priori expectations of populations in different regions being colonized at different times. Another possibility is that, even though we excluded candidate SNPs in these analyses, selection has a role in producing this underlying genetic structure. The genetic clusters approximately parallel three major geographical regions (lowland, plateau and mountain) each of which contains freshwater habitat with distinctive biophysical attributes. Population in mountainous regions along western Graham Island and on Moresby Island live in lakes characterized by limited littoral zones, high water clarity and usually co-inhabiting rainbow trout. In contrast, the north-east lowlands are an expansive low-elevation muskeg, containing dark acidic water and generally small shallow lakes. Central Graham Island contains lakes spanning the range of biophysical characters. Selection between environments can have effects across the genome, as demonstrated in stickleback (Hohenlohe *et al*. 2010; Jones *et al*. 2012b; Roesti *et al*. 2012) and other systems (Michel *et al*. 2010) and could contribute to the clustering especially if a larger proportion of the genome than currently expected is involved in local adaptation (see Michel *et al*. 2010).

### Implications for morphological evolution

The Haida Gwaii stickleback populations, although geographically close, encompass the species range of morphological variation including adult body size, lateral plate number, spine number and nuptial expression (Moodie & Reimchen 1976b; Reimchen *et al*. 1985; Reimchen 1994; Spoljaric & Reimchen 2007). Most of these traits have continuous distributions, and they often but not always covary, so defining distinct morphs is not possible. Here we have focused on extremes of the distribution for unarmoured populations with near complete loss of bony lateral plates and giant populations with the largest recorded body lengths. Unarmoured populations occur scattered across several basal branches of the SNP-based tree and are found in two of three PCA clusters. Convincing evidence that similar environments drive loss of lateral plates is the existence of groups of unarmoured populations that are geographically close (<750 m apart) but genetically distant. These unarmoured populations represent headwaters lakes from neighbouring watersheds and they cluster genetically alongside populations with more typical freshwater morphology within their own watersheds. The eight giant stickleback populations belong to seven different freshwater lineages, consistent with the watershed-specific origin proposed previously (Deagle *et al*. 2012). It is unclear whether the loss of lateral plates or gigantism has a common genetic basis across these watersheds, due to our relatively sparse genomic coverage and covariance between morphological traits. However, parallel population divergences in several genetically distinctive watersheds provides excellent opportunities for future high resolution genomic studies examining the genetic basis of these traits (or potential for phenotypic plasticity; see Leaver & Reimchen 2012) and to examine the concept of convergence and independence at a genomic level (see Elmer & Meyer 2011).

### Adaptive genomic regions

By genotyping candidate SNPs linked to adaptive alleles at particular loci, we were able to examine their geographic distribution in more detail than in previous studies that focussed on either broader genomic (Hohenlohe *et al*. 2010; Jones *et al*. 2012b) or geographic coverage (Jones *et al*. 2012a). Our data indicate that all low-plated Haida Gwaii stickleback contain the *EDA* low-plated allele (i.e. we did not identify exceptions, unlike a Japanese freshwater population described in Colosimo *et al*. 2005). Similarly, many of the other marine–freshwater adaptive alleles identified in globally distributed populations also are strongly divergent in Haida Gwaii populations confirming that the same global variants are repeatedly re-used on a regional scale. This parallel reuse of standing genetic variation is a common theme in studies considering the genetic basis of adaptation in the stickleback and other species (e.g. Dasmahapatra *et al*. 2012; Miller *et al*. 2012). However, unique regional adaptive changes may be common but undetected in the current analysis as we specifically examined broad patterns of divergence across populations and focused on known global outliers SNPs. Other stickleback studies considering local variants across marine–freshwater and stream-lake transitions indicate many strong nonparallel genetic divergences also occur (Hohenlohe *et al*. 2010; Deagle *et al*. 2012; Jones *et al*. 2012b; Roesti *et al*. 2012).

Our geographic survey of many globally adaptive marine–freshwater divergent alleles highlights some cases in which marine-like alleles are being retained in freshwater. One example is stickleback in Poque Lake. These low-plated lake fish with typical lake morphology cluster strongly with marine stickleback when considering SNPs normally divergent between marine and freshwater. The retention of marine-like alleles was also apparent in the few lakes with completely plated stickleback. These lakes exhibit a broad range of genetic variation (heterozygosity from 0.06 to 0.29) therefore depending on the lake it is possible either that typical freshwater alleles are absent due to a population bottleneck during colonization, or freshwater alleles could be rare due to recent gene flow with marine fish. These populations of freshwater stickleback containing many marine-like alleles are all located in mountainous regions along the western and southern parts of the archipelago. These habitats may also limit genetic adaptation due to the absence of low-gradient streams and large brackish estuaries, habitat features that may facilitate transport of adaptive freshwater alleles between watersheds via marine stickleback (Colosimo *et al*. 2005; Schluter & Conte 2009). The stickleback we sampled from estuarine populations generally contained a mixture of allelic forms at adaptive marine–freshwater loci, suggesting that interbreeding between marine and freshwater is common when suitable habitat is present.

An alternative to the possibility that marine-like alleles ending up in some lakes through historical processes is that natural selection may promote retention of these alleles in certain lakes. The shared biophysical characteristics of lakes with the most marine-like alleles could be taken as evidence for this (mentioned above; see also T. E. Reimchen, C. A. Bergstrom and P. Nosil 2013). The feasibility of this adaptive scenario is more directly supported by our geographic survey of two genomic regions that are outliers in both marine–freshwater and stream-lake transitions (see Deagle *et al*. 2012). Based on our data, giant lake fish in the original three stream-lake populations surveyed have retained marine-like alleles at these two loci even though the more common freshwater alleles at these loci are present in the watersheds (i.e. in the stream fish), and despite the giant lake fish not generally containing marine-like SNPs in other genomic regions. This parallel pattern strongly suggests that selection is driving retention of these particular marine-like alleles. Some outlier SNPs in one genomic region (chr4: 19.8 Mb) are also retained in several other lakes containing giant stickleback, although there is considerable variation in haplotypes at this genomic region between populations. It is possible that some of the marine-like SNPs we find retained in freshwater populations are in genomic regions flanking adaptive variants and they simply reflect hitchhiking of neutral markers (see Roesti *et al*. 2012). Only with full sequences from several populations, it will be possible to determine whether the marine-like regions identified by SNPs in the current survey carry the same adaptive variants as marine stickleback, whether these alleles represent unique freshwater variants, or whether some of these are common freshwater alleles flanked by marine-like regions.

## Conclusions

Our detailed geographic survey of genetic variation in threespine stickleback provides an unprecedented view of population structure in this model species. These data indicate that even on a regional scale divergent phenotypes found in different watersheds usually represent replicated evolutionary events. It is also apparent that globally distributed alleles suited to freshwater conditions have been repeatedly selected in Haida Gwaii freshwater populations. Furthermore, admixture between marine and freshwater fish appears frequent (based on the estuarine fish we sampled) indicating allelic recycling between watersheds via marine stickleback is probably common at additional locally selected loci. Decades of ecological research highlights the multiple layers of selection acting on these ecological and morphologically diverse stickleback populations. If suites of genetic changes underlie each layer of selection, untangling the resultant patterns of parallel and nonparallel changes will require high resolution genomic sequence data and detailed ecological information from many contrasting populations. The pristine habitats in which most Haida Gwaii stickleback populations exist combined with previous ecological work and the broad genetic survey presented here all indicate this will be an excellent system in which to advance our rapidly emerging view of the genetic basis of adaptation.

## Data accessibility

Genotype data at 1170 loci for all 467 individual stickleback included in the study and associated sample information available at DRYAD entry doi:10.5061/dryad.d08 h9. NCBI dbSNP reference numbers and genomic locations for all SNPs provided in Table S2 (Supporting information).

## Supporting information

Fig S1 Detailed maps showing populations from Sangan watershed, Masset Inlet and lakes with unarmoured fish in adjacent watersheds. The position of these populations in the SNP tree is also shown.

Fig S2 Neighbour-joining distance tree constructed as in Fig 2 but with poorly supported (50% bootstrap cut-off value) branches condensed to highlight the unresolved basal node and well-supported clusters at higher levels.

Fig S3 Details of PCA of evenly spaced SNPs. Includes PCA plot labelled with population names and weighting of SNPs.

Fig S4 Tree-based analysis of stickleback from two populations that each contain both divergent mtDNA lineages (ENA and Japan Sea).

Fig S5 Analysis of SNPs that were the most divergent between freshwater and marine/estuarine collected stickleback.

Table S1 List of collection locations and sample sizes.

Table S2 NCBI dbSNP reference numbers and genomic locations for genotyped SNPs.
