# Supplementary material for: Phylogeography and adaptation genetics of stickleback from the Haida Gwaii archipelago revealed using genome-wide single nucleotide polymorphism genotyping
Source: Mol Ecol. 2013 Mar 4;22(7):1917–32. doi: 10.1111/mec.12215 (PMC3604130; doi:10.1111/mec.12215)
Supplement: Fig S1 — Detailed maps showing populations from Sangan watershed, Masset Inlet and lakes with unarmoured fish in adjacent watersheds. The position of these populations in the SNP tree is also shown. [file mec0022-1917-sd1.pdf]

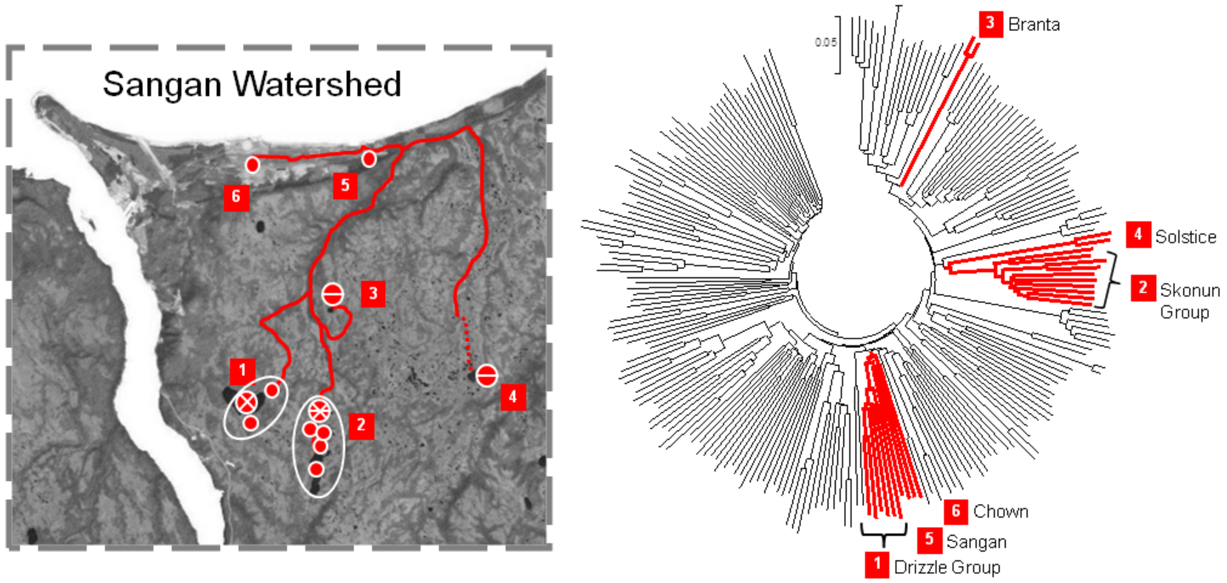

Fig. S1a Map of sites sampled in the Sangan watershed and their positions in the overall Haida Gwaii SNP tree. This watershed contains a great deal of morphological diversity including unarmoured (horizontal line through symbol) and giant stickleback (X through symbol; see main text and Reimchen *et al*. 1985 for details). The three clusters apparent in the tree are composed of six separate well supported groupings (based on those in the condensed tree with >50% bootstrap support; groups labelled). Drizzle Lake and adjoined streams group together (Group1) as do Skonun Lake, Skonun Outlet and neighbouring ponds (Group 2). Two isolated ponds (low heterozygosity) and two sites near the estuary (high heterozygosity) each form their own groups.

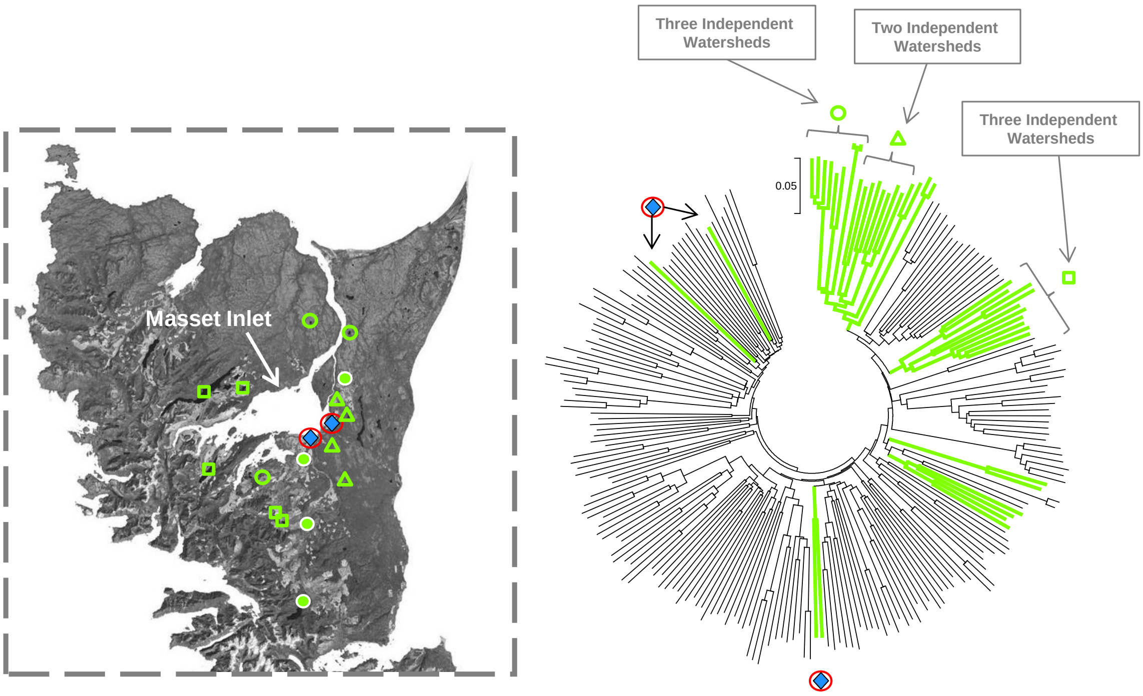

Fig. S1b Map of sites sampled from many watersheds which flow into Masset Inlet (a large marine inlet in central Graham Island) and their positions in the overall Haida Gwaii SNP tree. Three clusters (based on condensed tree with >50% bootstrap support) which join populations in separate drainages are labelled with open symbols. The open circles link three isolated populations, each in an independent watershed. The open triangles link four populations from two nearby watersheds. Finally the squares link populations from three additional watersheds. Estuarine sites (blue diamonds) from the area do not cluster with local watersheds. The linkages across freshwater drainages are likely the result of both low levels of ongoing gene flow and a common origin of marine populations in this area.

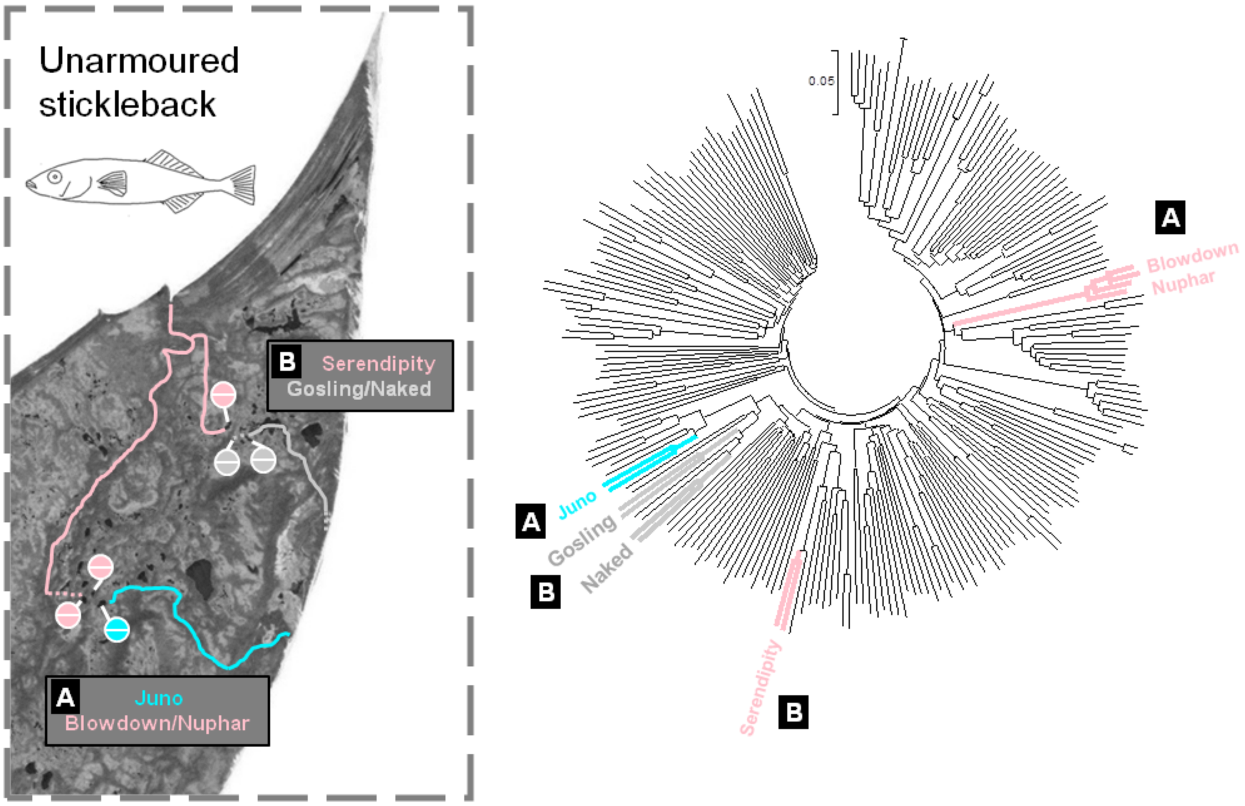

Fig. S1c Map of two areas with headwater lakes in adjacent watersheds containing unarmoured stickleback. In each case the unarmoured populations are very geographically close (< 750 m apart) but genetically distant as shown by their positions in the overall Haida Gwaii SNP tree.
