# Supplementary material for: Phylogeography and adaptation genetics of stickleback from the Haida Gwaii archipelago revealed using genome-wide single nucleotide polymorphism genotyping
Source: Mol Ecol. 2013 Mar 4;22(7):1917–32. doi: 10.1111/mec.12215 (PMC3604130; doi:10.1111/mec.12215)
Supplement: Fig S3 — Details of PCA of evenly spaced SNPs. Includes PCA plot labelled with population names and weighting of SNPs. [file mec0022-1917-sd3.pdf]

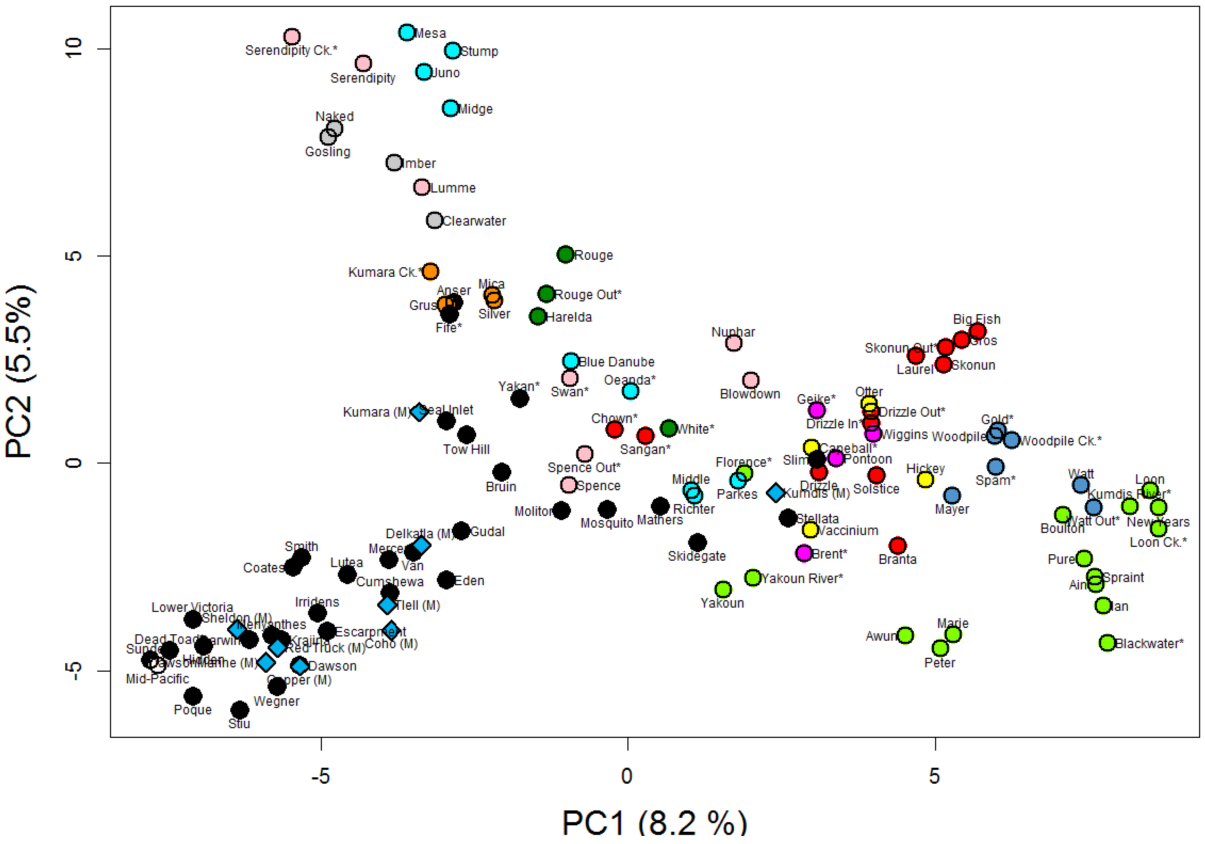

Fig. S3a Principal component analysis based on population SNP allele frequency data (evenly spaced SNPs; n=760). Each point is labelled with population name, colours follow scheme in Figure 1 with the mid-Pacific sample labelled white.

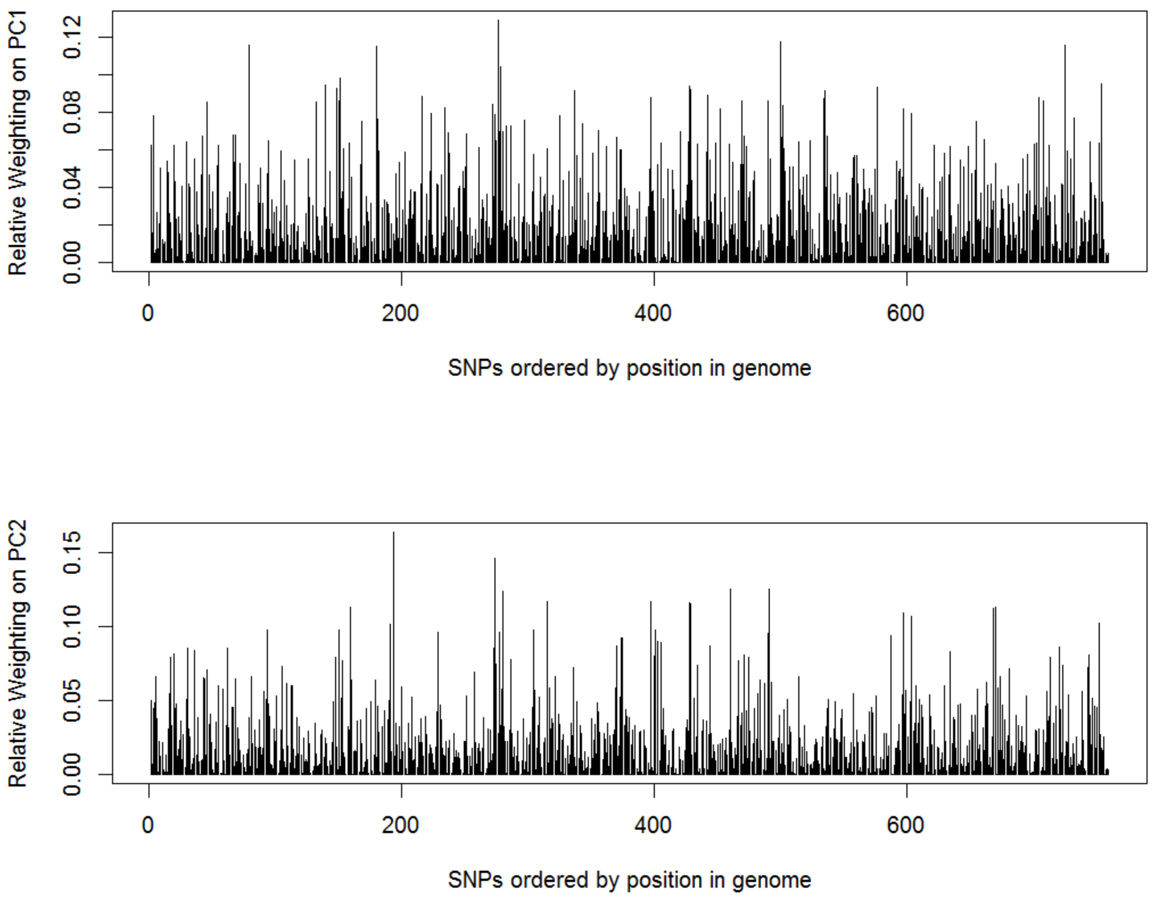

Fig. S3b Relative weighting of evenly spaced SNPs (n=760) on first two principal components. These plots indicate that weighting is spread out across many SNPs. To look at how many SNP were contributing to the observed groupings for each PC we ordered the 760 SNPs based on their relative weightings on the eigenvector. We then created 76 data subsets each consisting of 10 SNPs with successively lower weightings (e.g. $PC1_{SNP1-10}$, $PC1_{SNP11-20}$, $PC1_{SNP21-30}$, etc.). Next, the correlation between the PC based on all 760 SNPs and the first PC from each of the 10 SNP data subsets was examined. This allows us to see if the position on PC1 (based on all the data) is being maintained by the 10 SNP data subsets (see plots Fig. S3c-d).

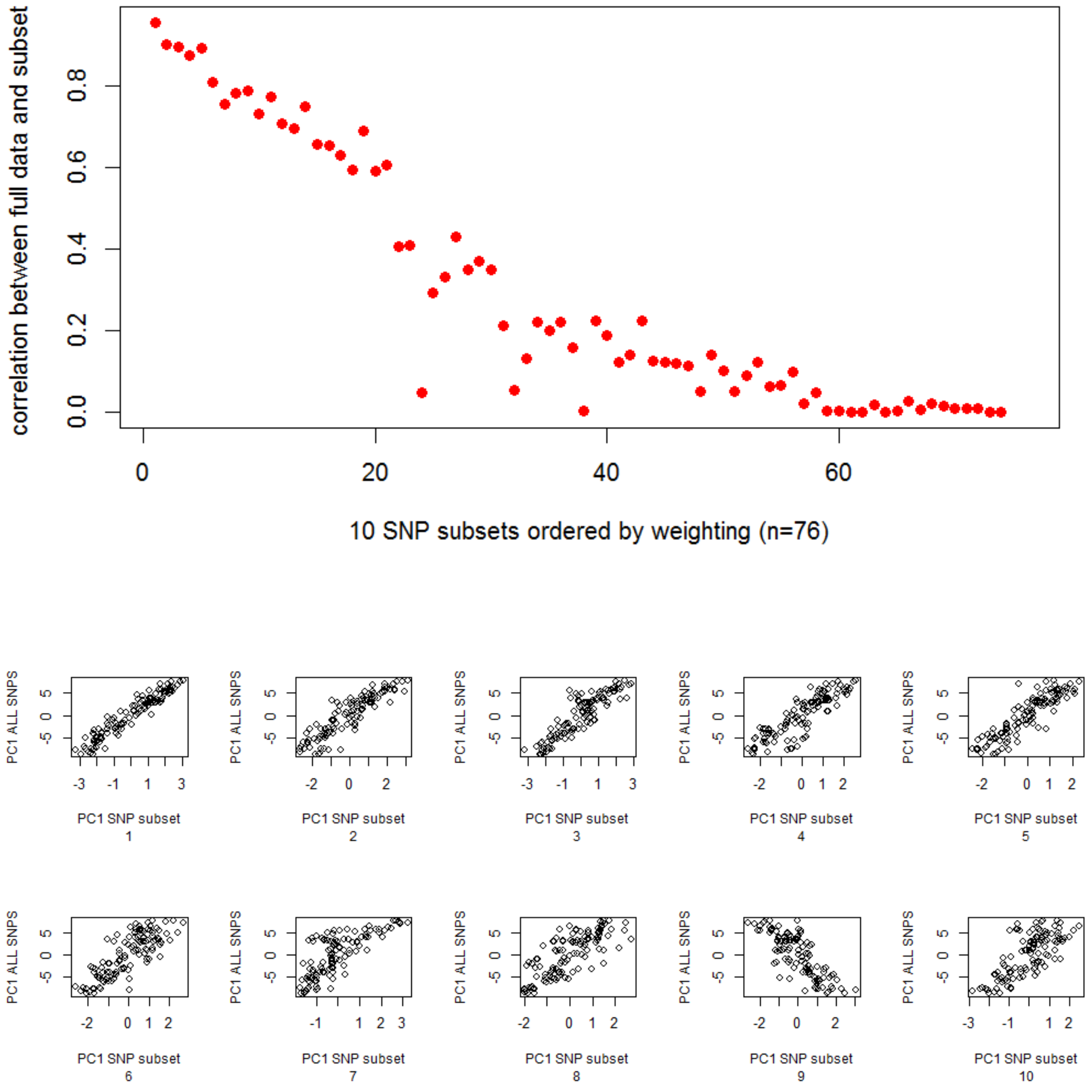

Fig. S3c These plots were used to determine how many SNPs were contributing to overall structure we see in Haida Gwaii Stickleback populations based on PCA. Top plot shows correlation between population PC1 scores (based on all 760 SNPs) with PC1 scores based on each of 76 data subsets. The data subsets each consisting of 10 SNPs with successively lower weightings ($PC1_{SNP1\text{-}10}$, $PC1_{SNP11\text{-}20}$, $PC1_{SNP21\text{-}30}$, etc.); see Fig. S3b for details. Lower plots show population PC1 score based on full dataset and population PC1 score based on the first 10 data subsets. These plots show that all subsets of these top 100 SNPs separate stickleback populations similarly to what we observe with whole dataset (i.e. many SNPs are contributing to the PC1 structuring).

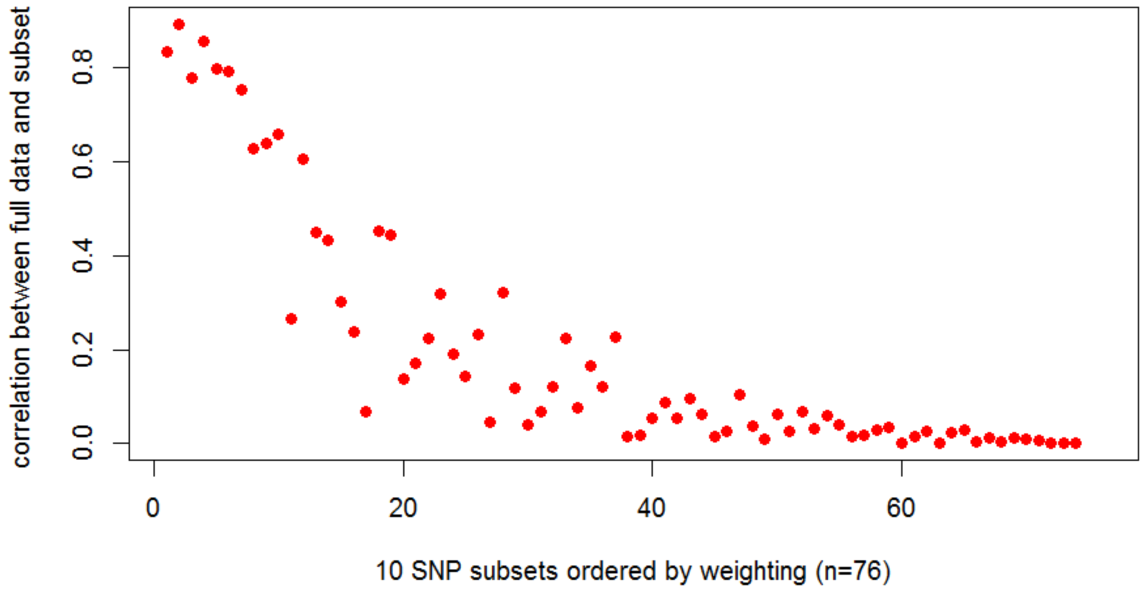

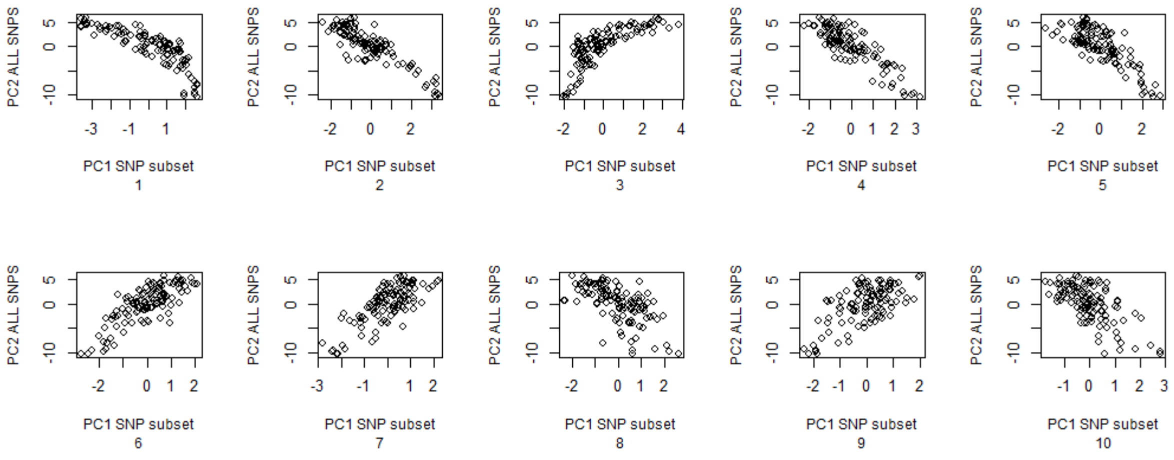

Fig. S3d These plots are the same as those in Fig S3c except the number of SNPs contributing to population separation on PC2 is being considered (see Fig S3c caption for details). Again these data show that all subsets of these top 100 SNPs separate stickleback populations similarly to what we observe with whole dataset (i.e. many SNPs are contributing to the PC2 structuring).
