# Supplementary material for: Phylogeography and adaptation genetics of stickleback from the Haida Gwaii archipelago revealed using genome-wide single nucleotide polymorphism genotyping
Source: Mol Ecol. 2013 Mar 4;22(7):1917–32. doi: 10.1111/mec.12215 (PMC3604130; doi:10.1111/mec.12215)
Supplement: Fig S4 — Tree-based analysis of stickleback from two populations that each contain both divergent mtDNA lineages (ENA and Japan Sea). [file mec0022-1917-sd4.pdf]

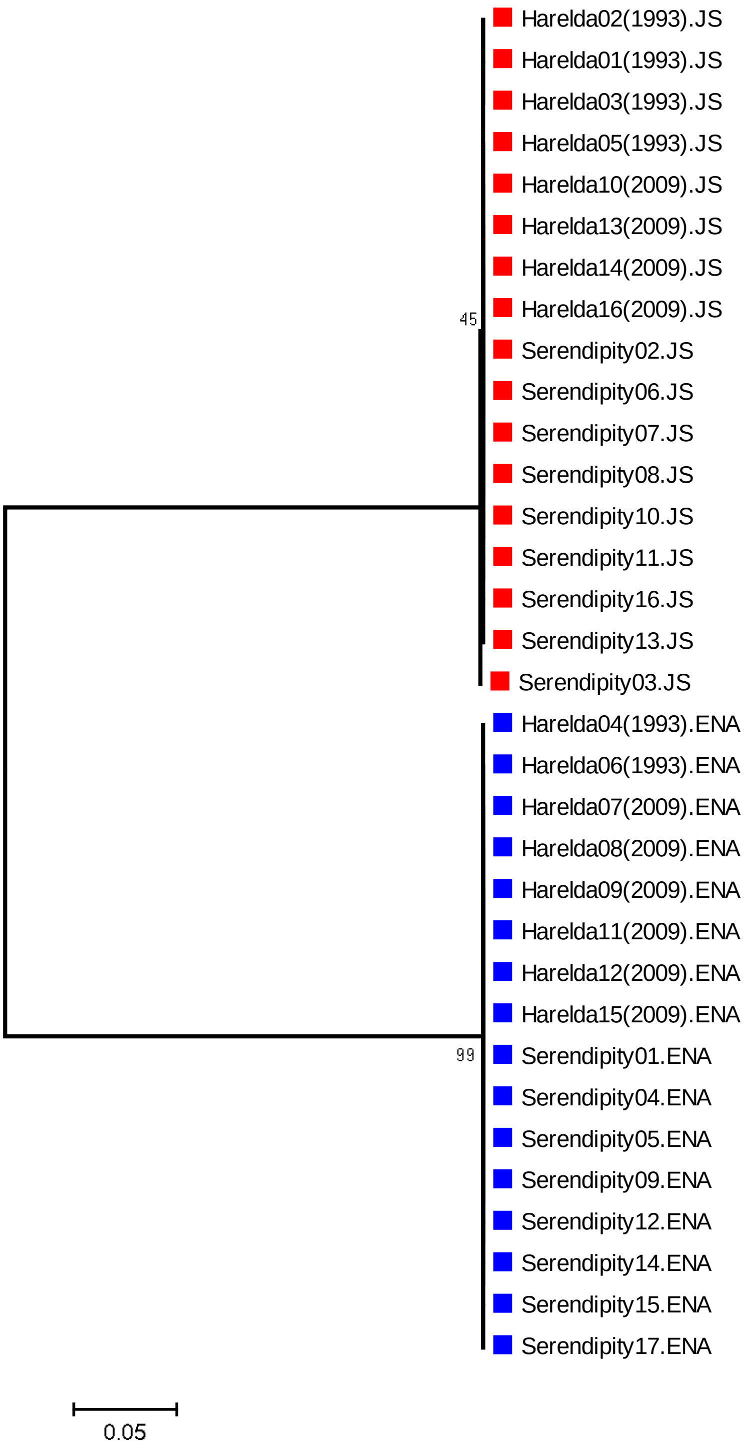

Fig. S4a Neighbour-joining distance tree of 33 stickleback in two lakes based on 10 mtDNA SNPs. Symbols show individuals belonging to JS (red) or ENA lineage (blue) mtDNA lineage. When analysis is only based on mtDNA these lakes both appear to contain two ancient stickleback genetic lineages. Bootstrap test percentages (1000 replicates) are shown next to the branches, distances were computed using p-distance

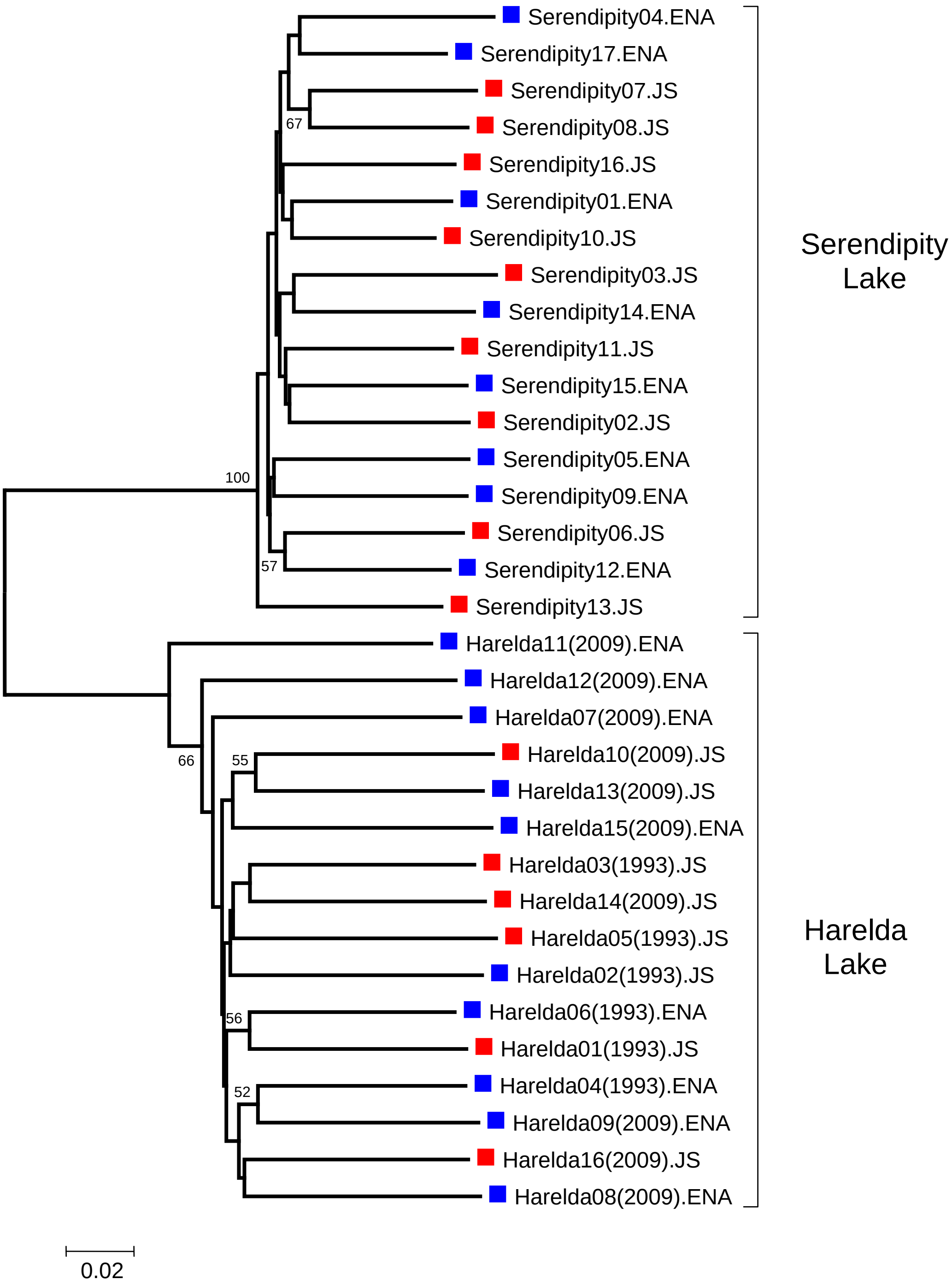

Fig. S4b Neighbour-joining distance tree of 33 stickleback in two lakes based on 760 nuclear SNPs. These lakes now separate by locality despite both containing two highly divergent mtDNA lineages. Symbols show JS (red) or ENA lineage (blue). Bootstrap test percentages (1000 replicates) are shown next to the branches, distances were computed using p-distance. The Harelda Lake samples included fish sampled in 1993 and 2009, the interspersion of these indicate sampling year had little effect on overall genotype.
