# Supplementary material for: Phylogeography and adaptation genetics of stickleback from the Haida Gwaii archipelago revealed using genome-wide single nucleotide polymorphism genotyping
Source: Mol Ecol. 2013 Mar 4;22(7):1917–32. doi: 10.1111/mec.12215 (PMC3604130; doi:10.1111/mec.12215)
Supplement: Fig S5 — Analysis of SNPs that were the most divergent between freshwater and marine/estuarine collected stickleback. [file mec0022-1917-sd5.pdf]

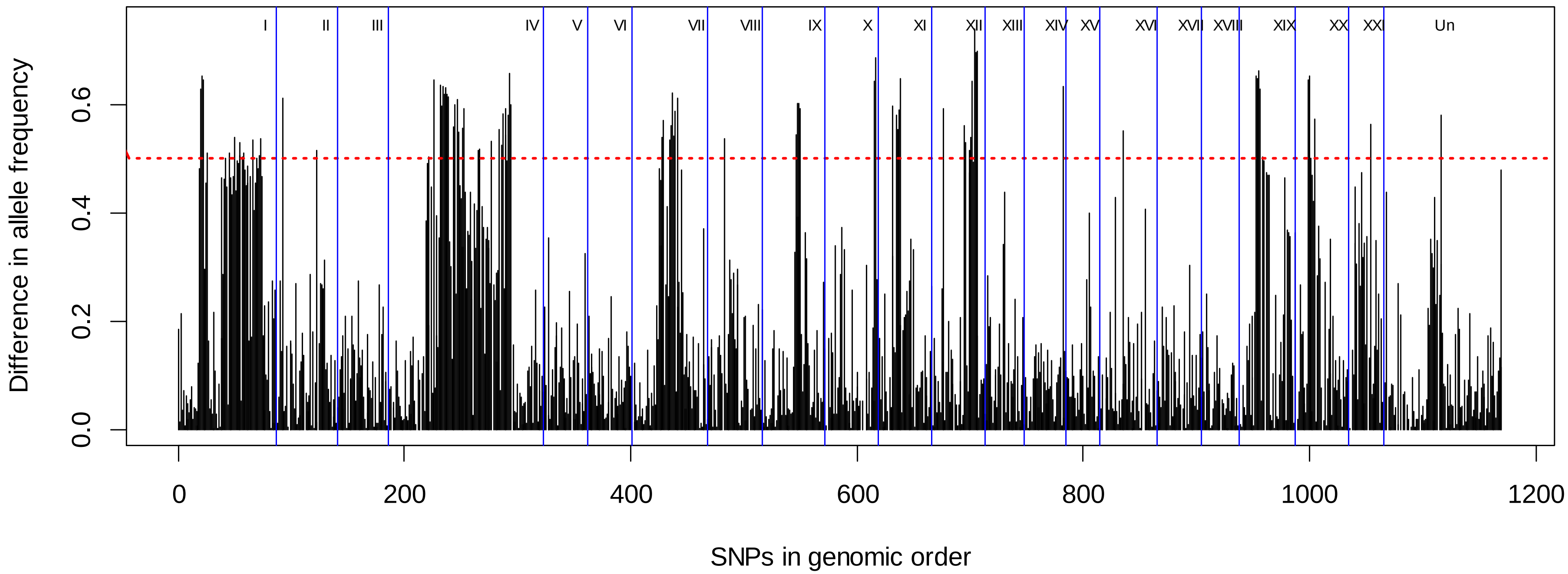

Fig. S5a Allele frequency differences between freshwater and marine/estuarine collection localities for SNP markers ordered across the genome (Vertical lines separate 21 chromosomes and unassembled scaffolds). Horizontal dotted line shows threshold used to define `habitat associated SNPs' used in PCA presented in figure 5b (these 86 SNPS are listed and genotypes shown in the figure below). Values calculated as allele frequency difference between 205 freshwater stickleback (two from each of 104 populations, except 3 populations with only single sample) and the 31 marine stickleback in our dataset.

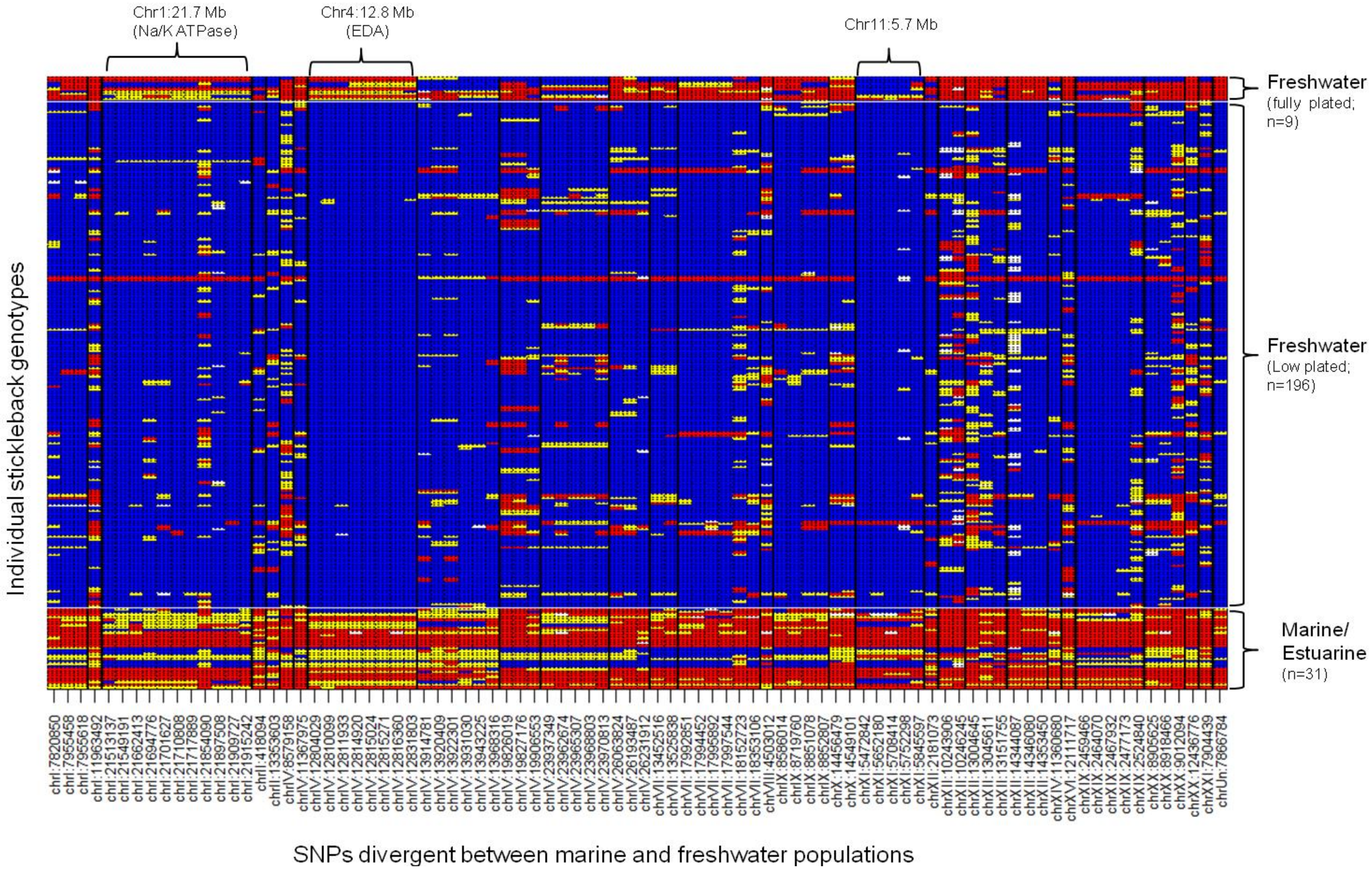

Fig. S5b Image plot showing individual stickleback genotypes for 86 SNPs that are strongly differentiated between freshwater and marine/estuarine collection localities (see Table S5 below). Genotypes were colour-coded so markers homozygous for the most common allele in marine samples are red (yellow is heterozygote, blue is homozygote for opposite allele). Samples from bottom to top: marine stickleback, low plated freshwater stickleback (two per population) and fully plated stickleback (two per population). Vertical line separate 28 potentially linked genomic regions tagged by multiple SNPs; some genomic regions mentioned in text are labelled at top.

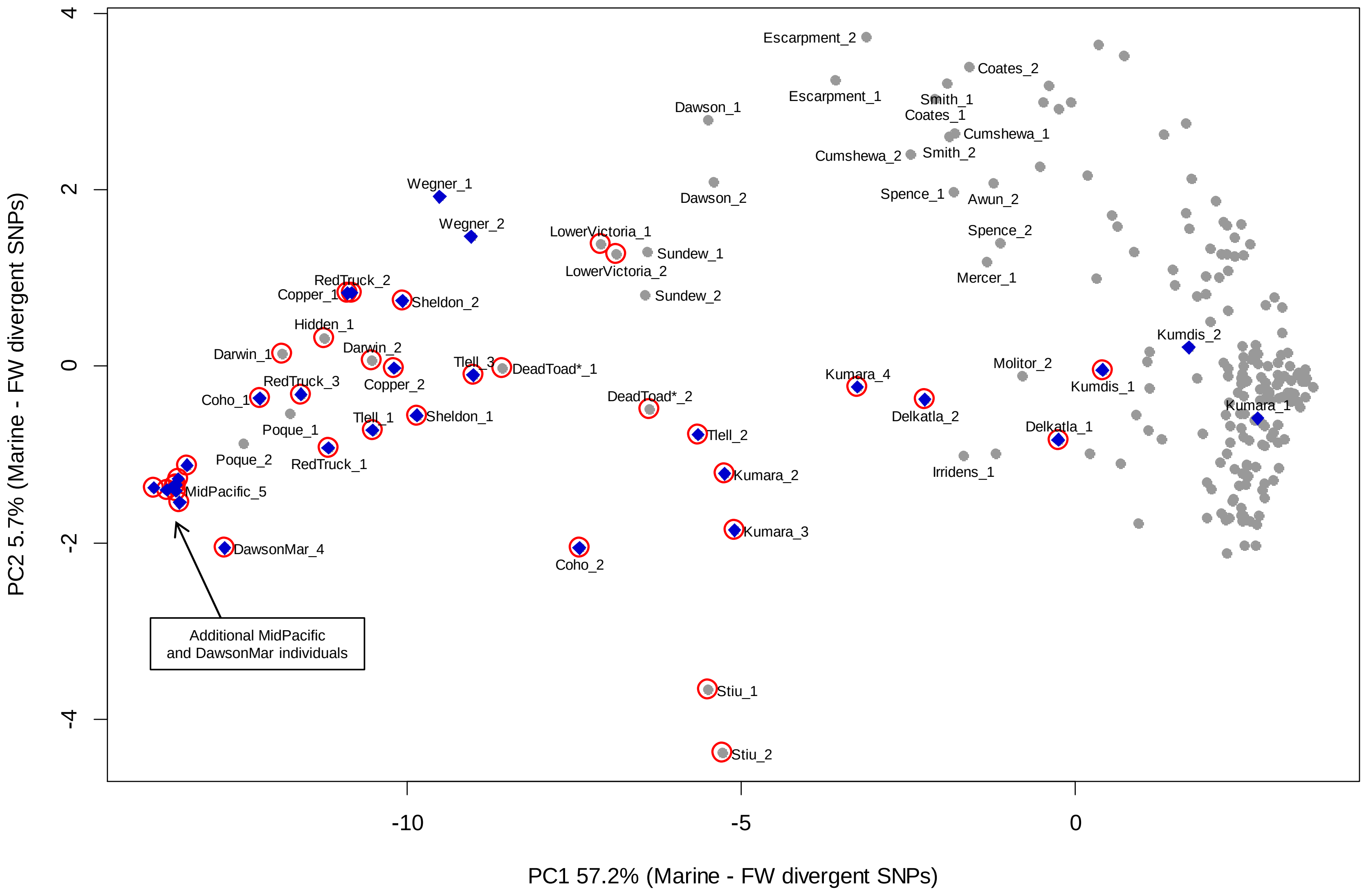

Fig. S5c PCA plot showing positioning of individual stickleback based on subset of SNPs most divergent in allele frequency between marine and freshwater localities (SNPs identified in Fig 5a; n=78 when SNPs linked to EDA are excluded). Stickleback from marine/estuarine collection sites are shown as blue diamonds, fully plated individuals are circled in red. The population of origin are labelled for marine, fully plated individuals and some of the more "marine-like" freshwater populations.

Table S5 List SNPs that diverged most in frequency between marine and freshwater localities in the current dataset (86 SNPs from 28 genomic regions) and comparison with results from genome scans carried out by Hohenlohe *et al*. (2010) and Jones *et al*. (2012b). Red text highlights regions not previously identified as outliers.

Region	Mar-FW Haida Gwaii	Hohenlohe et al 2010[1]	Jones et al 2012b[2]
1	chrI:7820850	Yes	No
1	chrI:7955458	Yes	No
1	chrI:7955618	Yes	No
2	chrI:11963492	Yes*	No
3	chrI:21513137	Yes	Yes
3	chrI:21549191	Yes	Yes
3	chrI:21662413	Yes	Yes
3	chrI:21694776	Yes	Yes
3	chrI:21701627	Yes	Yes
3	chrI:21710808	Yes	Yes
3	chrI:21717889	Yes	Yes
3	chrI:21854090	Yes	Yes
3	chrI:21897508	Yes	Yes
3	chrI:21909727	Yes	Yes
3	chrI:21915242	Yes	Yes
4	chrII:418094	No	Yes
5	chrII:13353603	Yes*	No
6	chrIV:8579158	No	Yes
7	chrIV:11367975	Yes	No
8	chrIV:12804029	Yes	Yes
8	chrIV:12810099	Yes	Yes
8	chrIV:12811933	Yes	Yes
8	chrIV:12814920	Yes	Yes
8	chrIV:12815024	Yes	Yes
8	chrIV:12815271	Yes	Yes
8	chrIV:12816360	Yes	Yes
8	chrIV:12831803	Yes	Yes
9	chrIV:13914781	No	Yes
9	chrIV:13920409	No	Yes
9	chrIV:13922301	No	Yes
9	chrIV:13931030	No	No
9	chrIV:13943225	No	Yes
9	chrIV:13968316	No	Yes
10	chrIV:19826019	Yes	Yes
10	chrIV:19827176	Yes	Yes
10	chrIV:19906553	Yes	Yes
11	chrIV:23937349	Yes	Yes
11	chrIV:23962674	Yes	Yes
11	chrIV:23965307	Yes	Yes
11	chrIV:23968803	Yes	Yes
11	chrIV:23970813	Yes	Yes
12	chrIV:26063824	Yes	Yes
12	chrIV:26193487	Yes	No
12	chrIV:26231912	Yes	No
13	chrVII:13452516	Yes	No
13	chrVII:13525838	Yes	No

14	chrVII:17992851	Yes	Yes
14	chrVII:17994452	Yes	Yes
14	chrVII:17995892	Yes	Yes
14	chrVII:17997544	Yes	Yes
14	chrVII:18152723	Yes	No
14	chrVII:18353106	Yes	No
15	chrVIII:4503012	No	No
16	chrIX:8586014	No	Yes
16	chrIX:8719760	No	No
16	chrIX:8851078	No	Yes
16	chrIX:8852807	No	Yes
17	chrX:14456479	No	No
17	chrX:14549101	No	No
18	chrXI:5472842	Yes	Yes
18	chrXI:5652180	Yes	Yes
18	chrXI:5708414	Yes	Yes
18	chrXI:5752298	Yes	Yes
18	chrXI:5845597	Yes	Yes
19	chrXII:2181073	No	Yes
20	chrXII:10243906	No	No
20	chrXII:10246245	No	No
21	chrXII:13004645	Yes	No
21	chrXII:13045611	Yes	No
21	chrXII:13151755	Yes	Yes
22	chrXII:14344087	Yes	Yes
22	chrXII:14346080	Yes	Yes
22	chrXII:14353450	Yes	No
23	chrXIV:11360680	No	Yes
24	chrXVI:12111717	No	No
25	chrXIX:2459466	Yes*	Yes
25	chrXIX:2464070	Yes*	Yes
25	chrXIX:2467932	Yes*	Yes
25	chrXIX:2477173	Yes*	Yes
25	chrXIX:2524840	Yes*	Yes
26	chrXX:8905625	Yes	No
26	chrXX:8918466	Yes	No
26	chrXX:9012094	Yes	No
27	chrXX:12436776	Yes	No
28	chrXXI:7904439	No	No
X	chrUn:7866784	X	X

[1] Indicates if Hohenlohe *et al*. (2010) found F_{ST} was significantly elevated at this genomic region ($p \geq 10^{-5}$) between the Alaskan oceanic and freshwater populations they examined. An asterisk indicates region is an outlier for some populations, but not overall oceanic-freshwater comparison.

[2] Indicates if Jones *et al*. (2012) identified these loci as outliers.
