# Supplementary material for: Phylogeography and adaptation genetics of stickleback from the Haida Gwaii archipelago revealed using genome-wide single nucleotide polymorphism genotyping
Source: Mol Ecol. 2013 Mar 4;22(7):1917–32. doi: 10.1111/mec.12215 (PMC3604130; doi:10.1111/mec.12215)
Supplement: Table S1 — List of collection locations and sample sizes. [file mec0022-1917-sd6.pdf]

Pop name	Habitat	CollectionDate	# Genotyped	Heterozygosity	Region
Ain	Lake	1993	2	0.1608	plateau
Anser	Lake	2009	2	0.2421	lowlands
Awun	Lake	2009	2	0.2579	plateau
Big Fish	Lake	2009	2	0.1833	lowlands
Blackwater*	Stream	1993	2	0.0020	plateau
Blowdown	Lake	2009	2	0.0503	lowlands
Blue Danube	Lake	1993	2	0.2912	lowlands
Boulton	Lake	2010	2	0.0893	lowlands
Branta	Lake	2009	4	0.0447	lowlands
Brent*	Stream	1993	2	0.0284	plateau
Bruin	Lake	1993	2	0.2163	lowlands
Capeball*	Stream	1993	2	0.2991	lowlands
Chown*	Stream	2009	2	0.3132	lowlands
Clearwater	Lake	1993	4	0.2809	lowlands
Coates	Lake	2009	2	0.1112	mountain
Coho (M)	Marine	1993	2	0.3236	plateau
Copper (M)	Marine	2009	2	0.3089	plateau
Cumshewa	Lake	2009	2	0.1466	plateau
Darwin	Lake	2009	4	0.2913	mountain
Dawson	Lake	2009	2	0.2142	mountain
DawsonMarine (M)	Marine	2009	4	0.3096	mountain
Dead Toad*	Stream	2009	2	0.0593	mountain
Delkatla (M)	Marine	1993	2	0.3138	lowlands
Drizzle In*	Stream	2010	9	0.2154	lowlands
Drizzle	Lake	2009/2010	19	0.2340	lowlands
Drizzle Out*	Stream	2009/2010	20	0.2240	lowlands
Eden	Lake	2010	2	0.1834	plateau
Escarpment	Lake	1993	4	0.2478	mountain
Florence*	Stream	2009	2	0.1236	plateau
Geike*	Stream	2009	2	0.2720	lowlands
Gold*	Stream	2009/2010	16	0.2289	lowlands
Gosling	Lake	2009	2	0.1621	lowlands
Gros	Lake	2009	2	0.1741	lowlands
Grus	Lake	2009	2	0.3013	lowlands
Gudal	Lake	1993	4	0.2884	mountain
Harelda	Lake	1993/2009	16	0.1820	lowlands
Hickey	Lake	1993	4	0.1792	lowlands
Hidden	Lake	2009	1	0.2082	mountain
Ian	Lake	1993	2	0.1723	plateau
Imber	Lake	2009	2	0.2265	lowlands
Irridens	Lake	2009	2	0.2416	mountain
Juno	Lake	2009	2	0.1477	lowlands
Krajina	Lake	2009	2	0.1096	mountain
Kumara (M)	Marine	2009	4	0.3427	lowlands
Kumara Ck.*	Stream	2009	2	0.2986	lowlands
Kumdis (M)	Marine	2009	2	0.2956	lowlands
Kumdis River*	Stream	2009	2	0.1690	lowlands
Laurel	Lake	1993	3	0.1852	lowlands
Loon Ck.*	Stream	2009	10	0.1515	lowlands

Loon	Lake	2009	4		0.1742	lowlands
Lower Victoria	Lake	1993	2		0.0563	mountain
Lumme	Lake	2009	2		0.2180	lowlands
Fife*	Stream	2009	2		0.2946	lowlands
Lutea	Lake	2009	2		0.2432	mountain
Marie	Lake	2009	2		0.1936	plateau
Mathers	Lake	2010	2		0.2037	mountain
Mayer#	Lake	2009/2010	18		0.2592	lowlands
Menyanthes	Lake	2009	2		0.1408	mountain
Mercer	Lake	2007	4		0.2927	mountain
Mesa	Lake	1993	2		0.1240	lowlands
Mica	Lake	2009	2		0.3050	lowlands
Middle	Lake	2009	2		0.0733	lowlands
Midge	Lake	1993	2		0.1886	lowlands
Molitor	Lake	2009	2		0.2360	plateau
Mosquito	Lake	2009	2		0.2659	plateau
Naked	Lake	2009	2		0.1726	lowlands
New Years	Lake	2009	2		0.1702	plateau
Nuphar	Lake	2009	2		0.1055	lowlands
Oeanda*	Stream	1993	2		0.2768	lowlands
Otter	Lake	1993	2		0.2693	lowlands
Parkes	Lake	2009	2		0.0940	lowlands
Peter	Lake	2009	2		0.1143	plateau
Pontoon	Lake	1993	2		0.2388	plateau
Poque	Lake	2009	2		0.1887	mountain
Pure	Lake	2009	2		0.1046	lowlands
Pure Out*#	Stream	2009	2	NA		lowlands
Red Truck (M)	Marine	2009	3		0.3134	lowlands
Richter	Lake	2009	2		0.0700	lowlands
Rouge	Lake	1993	4		0.1053	lowlands
Rouge Out*	Stream	1993	3		0.2223	lowlands
Sangan*	Stream	2009	2		0.2884	lowlands
Seal Inlet	Lake	2009	1		0.2520	mountain
Serendipity Ck.*	Stream	1993	1		0.1532	lowlands
Serendipity	Lake	2009	18		0.1410	lowlands
Sheldon (M)	Marine	2009	2		0.3153	plateau
Silver	Lake	2009	2		0.2768	lowlands
Skidegate	Lake	2009	2		0.2860	plateau
Skonun	Lake	2009	12		0.1910	lowlands
Skonun Out*	Stream	2009	10		0.1826	lowlands
Slim	Lake	1993	2		0.2126	lowlands
Smith	Lake	2009	2		0.1848	mountain
Solstice	Lake	2009	4		0.0859	lowlands
Spam*	Stream	2010	19		0.2313	lowlands
Spence	Lake	2009/2010	18		0.2340	lowlands
Spence Out*	Stream	2009/2010	17		0.2825	lowlands
Spraint	Lake	1993	2		0.1450	lowlands
Stellata	Lake	2010	2		0.0913	plateau
Stiu	Lake	2009	3		0.1506	mountain
Stump	Lake	1993	2		0.1499	lowlands

Sundew	Lake	2009	2	0.1418	mountain
Swan*	Stream	2009	2	0.2769	lowlands
Tlell (M)	Marine	2009	3	0.3325	lowlands
Tow Hill	Lake	2009	2	0.2903	lowlands
Vaccinium	Lake	2009	2	0.2445	lowlands
Van	Lake	2009	2	0.2376	mountain
Watt	Lake	2009	10	0.1293	lowlands
Watt Out*	Stream	2010	2	0.1309	lowlands
Wegner	Marine	2009	2	0.2892	mountain
White*	Stream	1993	2	0.2408	lowlands
Wiggins	Lake	1993	2	0.2170	lowlands
Woodpile	Lake	2009	10	0.2399	lowlands
Woodpile Ck.*	Stream	2009/2010	16	0.2318	lowlands
Yakan*	Stream	2009	2	0.2617	lowlands
Yakoun	Lake	2009	2	0.2568	plateau
Yakoun River*	Stream	2009	2	0.2485	plateau
			**462**		
mid-Pacific Ocean	Marine		5	0.2847	marine

These locations included stream samples collected within 5 meters downstream of the lake.
These stream samples (Pure Out and Mayer Out) were included on tree,
but excluded for most analyses.
* Stream collected samples
(M) = Marine/Estuary samples
