# Supplementary material for: Phylogeography and adaptation genetics of stickleback from the Haida Gwaii archipelago revealed using genome-wide single nucleotide polymorphism genotyping
Source: Mol Ecol. 2013 Mar 4;22(7):1917–32. doi: 10.1111/mec.12215 (PMC3604130; doi:10.1111/mec.12215)
Supplement: Table S2 — NCBI dbSNP reference numbers and genomic locations for genotyped SNPs. [file mec0022-1917-sd7.pdf]

SNP #	NamePos	Chr	SNP Group	NCBI dbSNP submitted SNP ss	NCBI dbSNP reference SNP rs
1	chrI:1550	chrI	Evenly Distributed	418641979	na
2	chrI:675926	chrI	Evenly Distributed	244222765	119103715
3	chrI:881806	chrI	Evenly Distributed	244222766	119103716
4	chrI:913033	chrI	Evenly Distributed	120258411	119103371
5	chrI:1245655	chrI	Evenly Distributed	418641981	na
6	chrI:1320011	chrI	Evenly Distributed	418641982	na
7	chrI:1498136	chrI	Candidate	418642693	na
8	chrI:1501000	chrI	Candidate	418642653	na
9	chrI:1549902	chrI	Evenly Distributed	244222767	119103717
10	chrI:2718044	chrI	Evenly Distributed	418641984	na
11	chrI:2823839	chrI	Evenly Distributed	418641985	na
12	chrI:3182664	chrI	Evenly Distributed	418641986	na
13	chrI:3310077	chrI	Evenly Distributed	244222768	119103718
14	chrI:3538018	chrI	Evenly Distributed	418641987	na
15	chrI:4171190	chrI	Evenly Distributed	244222769	119103719
16	chrI:4219350	chrI	Evenly Distributed	244222770	119103720
17	chrI:4387693	chrI	Evenly Distributed	418641988	na
18	chrI:4816374	chrI	Evenly Distributed	244222771	119103721
19	chrI:7818359	chrI	Candidate	418641994	na
20	chrI:7820850	chrI	Candidate	244222772	119103722
21	chrI:7955458	chrI	Candidate	120258413	119103373
22	chrI:7955618	chrI	Candidate	252841118	119104124
23	chrI:8040035	chrI	Candidate	418641995	na
24	chrI:8072483	chrI	Candidate	244222773	119103723
25	chrI:9345491	chrI	Candidate	120258414	119103374
26	chrI:11963492	chrI	Candidate	120258415	119103375
27	chrI:12038660	chrI	Candidate	120258416	119103376
28	chrI:12231274	chrI	Candidate	418641997	na
29	chrI:14261764	chrI	Evenly Distributed	418641998	na
30	chrI:14824378	chrI	Evenly Distributed	418641999	na
31	chrI:15145305	chrI	Evenly Distributed	418642000	na
32	chrI:15679433	chrI	Evenly Distributed	418642001	na
33	chrI:17306554	chrI	Evenly Distributed	418642003	na
34	chrI:18548172	chrI	Candidate	418642004	na
35	chrI:19946499	chrI	Evenly Distributed	418642005	na
36	chrI:20584613	chrI	Evenly Distributed	418642006	na
37	chrI:20740719	chrI	Evenly Distributed	418642007	na
38	chrI:21487034	chrI	Candidate	418642008	na
39	chrI:21495017	chrI	Candidate	244223004	119103954
40	chrI:21506736	chrI	Candidate	418642654	na
41	chrI:21511782	chrI	Candidate	244223005	119103955
42	chrI:21513137	chrI	Candidate	244223006	119103956
43	chrI:21535880	chrI	Candidate	244223007	119103957
44	chrI:21548191	chrI	Candidate	418642655	na
45	chrI:21549191	chrI	Candidate	244223008	119103958
46	chrI:21571342	chrI	Candidate	244223009	119103959
47	chrI:21593524	chrI	Candidate	244223010	119103960
48	chrI:21621170	chrI	Candidate	244223011	119103961
49	chrI:21648186	chrI	Candidate	244223013	119103963
50	chrI:21662413	chrI	Candidate	244223014	119103964
51	chrI:21672254	chrI	Candidate	244223015	119103965
52	chrI:21683350	chrI	Candidate	244223016	119103966

53	chrI:21689292	chrI	Candidate	244222775	119103725
54	chrI:21694776	chrI	Candidate	244223017	119103967
55	chrI:21701627	chrI	Candidate	244223018	119103968
56	chrI:21708011	chrI	Candidate	418642656	na
57	chrI:21710808	chrI	Candidate	244223019	119103969
58	chrI:21717889	chrI	Candidate	244223021	119103971
59	chrI:21719286	chrI	Candidate	244223022	119103972
60	chrI:21724794	chrI	Candidate	244223023	119103973
61	chrI:21762138	chrI	Candidate	244223024	119103974
62	chrI:21773142	chrI	Candidate	244223025	119103975
63	chrI:21805675	chrI	Candidate	244223026	119103976
64	chrI:21836364	chrI	Candidate	244223028	119103978
65	chrI:21850057	chrI	Candidate	244223029	119103979
66	chrI:21854090	chrI	Candidate	244223030	119103980
67	chrI:21859778	chrI	Candidate	244223031	119103981
68	chrI:21883499	chrI	Candidate	244223032	119103982
69	chrI:21897508	chrI	Candidate	244223033	119103983
70	chrI:21899980	chrI	Candidate	244223034	119103984
71	chrI:21908211	chrI	Candidate	244223035	119103985
72	chrI:21909727	chrI	Candidate	244223036	119103986
73	chrI:21915242	chrI	Candidate	244223037	119103987
74	chrI:21929549	chrI	Candidate	244223038	119103988
75	chrI:21951727	chrI	Candidate	252841103	119104109
76	chrI:22164046	chrI	Candidate	418642009	na
77	chrI:22336815	chrI	Candidate	418642694	na
78	chrI:22348583	chrI	Candidate	418642657	na
79	chrI:22361077	chrI	Evenly Distributed	120258417	119103377
80	chrI:22716347	chrI	Evenly Distributed	418642010	na
81	chrI:23569502	chrI	Evenly Distributed	244222776	119103726
82	chrI:24983706	chrI	Evenly Distributed	418642012	na
83	chrI:25201193	chrI	Evenly Distributed	252841111	119104117
84	chrI:25560380	chrI	Evenly Distributed	418642013	na
85	chrI:26879230	chrI	Evenly Distributed	244222777	119103727
86	chrI:27642534	chrI	Evenly Distributed	418642015	na
87	chrI:27721035	chrI	Evenly Distributed	244222778	119103728
88	chrII:213137	chrII	Evenly Distributed	418642016	na
89	chrII:377479	chrII	Evenly Distributed	244222779	119103729
90	chrII:409087	chrII	Candidate	244223040	119103990
91	chrII:409407	chrII	Candidate	418642695	na
92	chrII:413850	chrII	Candidate	418642658	na
93	chrII:418094	chrII	Candidate	244222780	119103730
94	chrII:491352	chrII	Evenly Distributed	418642017	na
95	chrII:533883	chrII	Evenly Distributed	120258418	119103378
96	chrII:640670	chrII	Evenly Distributed	418642019	na
97	chrII:1502763	chrII	Evenly Distributed	418642020	na
98	chrII:1654791	chrII	Evenly Distributed	418642021	na
99	chrII:1902367	chrII	Evenly Distributed	418642022	na
100	chrII:2829800	chrII	Evenly Distributed	252841149	119104155
101	chrII:3131035	chrII	Evenly Distributed	252841121	119104127
102	chrII:3384330	chrII	Evenly Distributed	120258421	119103381
103	chrII:3931852	chrII	Evenly Distributed	418642025	na
104	chrII:4157699	chrII	Evenly Distributed	252841112	119104118
105	chrII:4530808	chrII	Evenly Distributed	120258423	119103383
106	chrII:5111258	chrII	Evenly Distributed	418642028	na
107	chrII:5216711	chrII	Evenly Distributed	418642029	na
108	chrII:5590307	chrII	Evenly Distributed	252841076	119104082

109	chrII:5914538	chrII	Assembly	418642030	na
110	chrII:5935944	chrII	Assembly	252841148	119104154
111	chrII:6064631	chrII	Assembly	418641916	na
112	chrII:6300744	chrII	Evenly Distributed	120258424	119103384
113	chrII:6475468	chrII	Evenly Distributed	244222782	119103732
114	chrII:8246178	chrII	Assembly	418642032	na
115	chrII:8305286	chrII	Evenly Distributed	418642033	na
116	chrII:8979491	chrII	Evenly Distributed	252841135	119104141
117	chrII:10092618	chrII	Evenly Distributed	418642034	na
118	chrII:11273903	chrII	Evenly Distributed	418642035	na
119	chrII:11979040	chrII	Candidate	418642036	na
120	chrII:12116321	chrII	Evenly Distributed	418642037	na
121	chrII:12292176	chrII	Evenly Distributed	120258425	119103385
122	chrII:13353603	chrII	Candidate	244222783	119103733
123	chrII:14155271	chrII	Evenly Distributed	418642038	na
124	chrII:14586111	chrII	Evenly Distributed	418642039	na
125	chrII:14611516	chrII	Evenly Distributed	244222784	119103734
126	chrII:14804177	chrII	Candidate	418642659	na
127	chrII:14805042	chrII	Candidate	418642696	na
128	chrII:14807513	chrII	Candidate	418642660	na
129	chrII:14991358	chrII	Evenly Distributed	418642040	na
130	chrII:17312835	chrII	Evenly Distributed	418642041	na
131	chrII:18276031	chrII	Evenly Distributed	418642043	na
132	chrII:19324477	chrII	Evenly Distributed	418642044	na
133	chrII:19421318	chrII	Evenly Distributed	418642045	na
134	chrII:19985741	chrII	Evenly Distributed	244222785	119103735
135	chrII:20376697	chrII	Evenly Distributed	418642050	na
136	chrII:20425752	chrII	Evenly Distributed	418642051	na
137	chrII:20714675	chrII	Evenly Distributed	252841046	119104052
138	chrII:21013052	chrII	Evenly Distributed	418642052	na
139	chrII:21231538	chrII	Evenly Distributed	244222786	119103736
140	chrII:21379816	chrII	Evenly Distributed	418642053	na
141	chrII:22443700	chrII	Evenly Distributed	244222787	119103737
142	chrIII:105665	chrIII	Evenly Distributed	418642055	na
143	chrIII:639237	chrIII	Evenly Distributed	418642059	na
144	chrIII:706165	chrIII	Evenly Distributed	244222789	119103739
145	chrIII:1198125	chrIII	Evenly Distributed	120258428	119103388
146	chrIII:1651721	chrIII	Evenly Distributed	252841079	119104085
147	chrIII:1925911	chrIII	Evenly Distributed	418642062	na
148	chrIII:1968625	chrIII	Evenly Distributed	418642063	na
149	chrIII:2309984	chrIII	Evenly Distributed	418642064	na
150	chrIII:2376699	chrIII	Evenly Distributed	418642065	na
151	chrIII:2471292	chrIII	Evenly Distributed	418642066	na
152	chrIII:3156991	chrIII	Evenly Distributed	418642068	na
153	chrIII:3881955	chrIII	Evenly Distributed	418641917	na
154	chrIII:10376395	chrIII	Evenly Distributed	244222790	119103740
155	chrIII:11202761	chrIII	Evenly Distributed	418642070	na
156	chrIII:11302839	chrIII	Evenly Distributed	418642071	na
157	chrIII:11836494	chrIII	Evenly Distributed	418642072	na
158	chrIII:12316694	chrIII	Evenly Distributed	252841106	119104112
159	chrIII:12383383	chrIII	Evenly Distributed	418642073	na
160	chrIII:12433574	chrIII	Evenly Distributed	418642074	na
161	chrIII:12930427	chrIII	Evenly Distributed	418642076	na
162	chrIII:13397314	chrIII	Evenly Distributed	418642078	na
163	chrIII:13520975	chrIII	Evenly Distributed	252841102	119104108
164	chrIII:13582181	chrIII	Evenly Distributed	244222791	119103741

165	chrIII:13699701	chrIII	Evenly Distributed	418642079	na
166	chrIII:13911180	chrIII	Evenly Distributed	418642080	na
167	chrIII:13929118	chrIII	Evenly Distributed	244222792	119103742
168	chrIII:14048561	chrIII	Evenly Distributed	252841058	119104064
169	chrIII:14135608	chrIII	Evenly Distributed	418642081	na
170	chrIII:14248039	chrIII	Evenly Distributed	418642083	na
171	chrIII:14307973	chrIII	Evenly Distributed	244222793	119103743
172	chrIII:14393183	chrIII	Evenly Distributed	418642084	na
173	chrIII:14456990	chrIII	Evenly Distributed	252841063	119104069
174	chrIII:14690970	chrIII	Evenly Distributed	418641895	na
175	chrIII:14791668	chrIII	Evenly Distributed	418642086	na
176	chrIII:14892994	chrIII	Evenly Distributed	244222794	119103744
177	chrIII:15157782	chrIII	Evenly Distributed	418642087	na
178	chrIII:15185662	chrIII	Evenly Distributed	418642088	na
179	chrIII:15783657	chrIII	Evenly Distributed	252841104	119104110
180	chrIII:15793968	chrIII	Assembly	418642089	na
181	chrIII:15856499	chrIII	Evenly Distributed	120258429	119103389
182	chrIII:15933767	chrIII	Evenly Distributed	244222795	119103745
183	chrIII:16123944	chrIII	Evenly Distributed	418642090	na
184	chrIII:16224572	chrIII	Evenly Distributed	120258430	119103390
185	chrIII:16251071	chrIII	Evenly Distributed	120258431	119103391
186	chrIII:16463929	chrIII	Evenly Distributed	244222796	119103746
187	chrIV:219384	chrIV	Evenly Distributed	418642093	na
188	chrIV:339710	chrIV	Evenly Distributed	418642094	na
189	chrIV:428725	chrIV	Evenly Distributed	418642095	na
190	chrIV:707934	chrIV	Candidate	418642661	na
191	chrIV:743822	chrIV	Evenly Distributed	418641918	na
192	chrIV:1020296	chrIV	Evenly Distributed	252841071	119104077
193	chrIV:1121404	chrIV	Evenly Distributed	418642096	na
194	chrIV:1541964	chrIV	Evenly Distributed	244222797	119103747
195	chrIV:1595013	chrIV	Evenly Distributed	418642097	na
196	chrIV:1738651	chrIV	Evenly Distributed	418642098	na
197	chrIV:1942641	chrIV	Evenly Distributed	244222798	119103748
198	chrIV:2045971	chrIV	Evenly Distributed	418642099	na
199	chrIV:2274657	chrIV	Evenly Distributed	418642100	na
200	chrIV:2334501	chrIV	Evenly Distributed	418642101	na
201	chrIV:2544711	chrIV	Evenly Distributed	418642102	na
202	chrIV:2858360	chrIV	Evenly Distributed	252841155	119104161
203	chrIV:3334208	chrIV	Evenly Distributed	418642103	na
204	chrIV:3385310	chrIV	Evenly Distributed	418642104	na
205	chrIV:3764773	chrIV	Evenly Distributed	252841050	119104056
206	chrIV:4034002	chrIV	Evenly Distributed	120258432	119103392
207	chrIV:4065598	chrIV	Evenly Distributed	244222799	119103749
208	chrIV:4186582	chrIV	Evenly Distributed	418642105	na
209	chrIV:4310010	chrIV	Evenly Distributed	418642106	na
210	chrIV:4370951	chrIV	Evenly Distributed	418642107	na
211	chrIV:4437011	chrIV	Evenly Distributed	418642108	na
212	chrIV:4599765	chrIV	Evenly Distributed	244222800	119103750
213	chrIV:4787498	chrIV	Evenly Distributed	418642110	na
214	chrIV:5249216	chrIV	Evenly Distributed	418642112	na
215	chrIV:5290789	chrIV	Assembly	418642113	na
216	chrIV:5313693	chrIV	Evenly Distributed	120258433	119103393
217	chrIV:6128193	chrIV	Evenly Distributed	418642116	na
218	chrIV:6239168	chrIV	Assembly	418642118	na
219	chrIV:8545605	chrIV	Evenly Distributed	418642119	na
220	chrIV:8576794	chrIV	Candidate	244223068	119104018

221	chrIV:8579158	chrIV	Candidate	244223069	119104019
222	chrIV:9220132	chrIV	Assembly	418642120	na
223	chrIV:9309735	chrIV	Candidate	418642121	na
224	chrIV:10960835	chrIV	Candidate	120258434	119103394
225	chrIV:10997988	chrIV	Evenly Distributed	244222801	119103751
226	chrIV:11367975	chrIV	Evenly Distributed	120258435	119103395
227	chrIV:11926965	chrIV	Assembly	418642122	na
228	chrIV:12005099	chrIV	Candidate	418642662	na
229	chrIV:12005323	chrIV	Candidate	418642697	na
230	chrIV:12005556	chrIV	Candidate	252841179	119104185
231	chrIV:12022250	chrIV	Assembly	418642123	na
232	chrIV:12804029	chrIV	Candidate	244223043	119103993
233	chrIV:12810099	chrIV	Candidate	244223044	119103994
234	chrIV:12811933	chrIV	Candidate	244222802	119103752
235	chrIV:12814920	chrIV	Candidate	244223045	119103995
236	chrIV:12815024	chrIV	Candidate	244222803	119103753
237	chrIV:12815271	chrIV	Candidate	244223046	119103996
238	chrIV:12816360	chrIV	Candidate	244223047	119103997
239	chrIV:12831803	chrIV	Candidate	418642698	na
240	chrIV:12840362	chrIV	Candidate	418642663	na
241	chrIV:12955826	chrIV	Evenly Distributed	120258436	119103396
242	chrIV:13850026	chrIV	Candidate	244223049	119103999
243	chrIV:13914418	chrIV	Candidate	244223050	119104000
244	chrIV:13914781	chrIV	Candidate	244223052	119104002
245	chrIV:13920409	chrIV	Candidate	244223053	119104003
246	chrIV:13920868	chrIV	Candidate	418642664	na
247	chrIV:13922301	chrIV	Candidate	244223054	119104004
248	chrIV:13931030	chrIV	Candidate	418642124	na
249	chrIV:13940084	chrIV	Candidate	418642699	na
250	chrIV:13941508	chrIV	Candidate	418642665	na
251	chrIV:13943225	chrIV	Candidate	418642700	na
252	chrIV:13947621	chrIV	Candidate	418642666	na
253	chrIV:13968316	chrIV	Candidate	418642701	na
254	chrIV:13970860	chrIV	Candidate	418642667	na
255	chrIV:14325385	chrIV	Candidate	252841061	119104067
256	chrIV:15052901	chrIV	Evenly Distributed	244222804	119103754
257	chrIV:15530121	chrIV	Evenly Distributed	244222805	119103755
258	chrIV:15721538	chrIV	Assembly	244222806	119103756
259	chrIV:15737291	chrIV	Assembly	244222807	119103757
260	chrIV:18425274	chrIV	Evenly Distributed	120258437	119103397
261	chrIV:19271805	chrIV	Evenly Distributed	418642126	na
262	chrIV:19812956	chrIV	Candidate	244223055	119104005
263	chrIV:19814842	chrIV	Candidate	244223056	119104006
264	chrIV:19819889	chrIV	Candidate	244223057	119104007
265	chrIV:19826019	chrIV	Candidate	244223058	119104008
266	chrIV:19827176	chrIV	Candidate	244223059	119104009
267	chrIV:19856347	chrIV	Candidate	244223060	119104010
268	chrIV:19856750	chrIV	Candidate	418642668	na
269	chrIV:19863404	chrIV	Candidate	244223062	119104012
270	chrIV:19872201	chrIV	Candidate	418642669	na
271	chrIV:19872520	chrIV	Candidate	418642702	na
272	chrIV:19881291	chrIV	Candidate	418642670	na
273	chrIV:19881370	chrIV	Candidate	418642703	na
274	chrIV:19881515	chrIV	Candidate	418642671	na
275	chrIV:19890632	chrIV	Candidate	418642704	na
276	chrIV:19896811	chrIV	Candidate	244223063	119104013

277	chrIV:19906553	chrIV	Candidate	244223064	119104014
278	chrIV:21232476	chrIV	Evenly Distributed	418642127	na
279	chrIV:21605258	chrIV	Evenly Distributed	252841082	119104088
280	chrIV:22070002	chrIV	Evenly Distributed	418642128	na
281	chrIV:23711481	chrIV	Evenly Distributed	120258438	119103398
282	chrIV:23804450	chrIV	Candidate	120258439	119103399
283	chrIV:23809335	chrIV	Candidate	418642129	na
284	chrIV:23937349	chrIV	Evenly Distributed	120258440	119103400
285	chrIV:23953087	chrIV	Candidate	418642705	na
286	chrIV:23962674	chrIV	Candidate	418642672	na
287	chrIV:23965307	chrIV	Candidate	244223065	119104015
288	chrIV:23968037	chrIV	Candidate	418642673	na
289	chrIV:23968803	chrIV	Candidate	244223066	119104016
290	chrIV:23970813	chrIV	Candidate	252841168	119104174
291	chrIV:23979425	chrIV	Candidate	418642706	na
292	chrIV:26063824	chrIV	Candidate	244223067	119104017
293	chrIV:26193487	chrIV	Candidate	244222808	119103758
294	chrIV:26231912	chrIV	Candidate	418642131	na
295	chrIV:26692463	chrIV	Evenly Distributed	418641919	na
296	chrIV:27614532	chrIV	Evenly Distributed	120258442	119103402
297	chrIV:28999167	chrIV	Candidate	418642132	na
298	chrIV:29034665	chrIV	Evenly Distributed	418642133	na
299	chrIV:29563533	chrIV	Evenly Distributed	418642135	na
300	chrIV:29763654	chrIV	Evenly Distributed	120258443	119103403
301	chrIV:30384878	chrIV	Evenly Distributed	418642136	na
302	chrIV:30568387	chrIV	Evenly Distributed	252841083	119104089
303	chrIV:31166790	chrIV	Evenly Distributed	418642138	na
304	chrIV:31350187	chrIV	Evenly Distributed	418642140	na
305	chrIV:31406437	chrIV	Evenly Distributed	418642141	na
306	chrIV:31486885	chrIV	Evenly Distributed	418642142	na
307	chrIV:31583885	chrIV	Evenly Distributed	252841078	119104084
308	chrIV:31611147	chrIV	Evenly Distributed	252841084	119104090
309	chrIV:31740478	chrIV	Evenly Distributed	244222809	119103759
310	chrIV:32005807	chrIV	Evenly Distributed	120258445	119103405
311	chrIV:32033500	chrIV	Evenly Distributed	244222810	119103760
312	chrIV:32049547	chrIV	Evenly Distributed	418642143	na
313	chrIV:32092919	chrIV	Evenly Distributed	252841132	119104138
314	chrIV:32133665	chrIV	Evenly Distributed	244222811	119103761
315	chrIV:32148857	chrIV	Evenly Distributed	418642144	na
316	chrIV:32208230	chrIV	Evenly Distributed	418641896	na
317	chrIV:32277841	chrIV	Evenly Distributed	418642146	na
318	chrIV:32332380	chrIV	Evenly Distributed	418642147	na
319	chrIV:32350814	chrIV	Evenly Distributed	418642148	na
320	chrIV:32387818	chrIV	Evenly Distributed	120258447	119103407
321	chrIV:32487875	chrIV	Evenly Distributed	244222812	119103762
322	chrIV:32568140	chrIV	Evenly Distributed	418641897	na
323	chrIV:32592491	chrIV	Evenly Distributed	418642150	na
324	chrV:1238066	chrV	Evenly Distributed	120258448	119103408
325	chrV:1727383	chrV	Evenly Distributed	418642153	na
326	chrV:1815933	chrV	Assembly	418642154	na
327	chrV:2489551	chrV	Candidate	244223074	119104024
328	chrV:2489835	chrV	Candidate	244223075	119104025
329	chrV:2528528	chrV	Evenly Distributed	244222814	119103764
330	chrV:4574523	chrV	Candidate	418642157	na
331	chrV:4819972	chrV	Assembly	418642158	na
332	chrV:4852958	chrV	Assembly	244222815	119103765

333	chrV:5064057	chrV	Evenly Distributed	418642160	na
334	chrV:6848817	chrV	Assembly	418641898	na
335	chrV:7791830	chrV	Evenly Distributed	252841093	119104099
336	chrV:8211082	chrV	Candidate	252841163	119104169
337	chrV:8214190	chrV	Candidate	252841172	119104178
338	chrV:8327818	chrV	Evenly Distributed	244222816	119103766
339	chrV:8562218	chrV	Evenly Distributed	244222817	119103767
340	chrV:8828019	chrV	Evenly Distributed	418642161	na
341	chrV:9092208	chrV	Evenly Distributed	120258450	119103410
342	chrV:9157076	chrV	Evenly Distributed	244222818	119103768
343	chrV:9219138	chrV	Evenly Distributed	120258451	119103411
344	chrV:9521704	chrV	Evenly Distributed	418642162	na
345	chrV:9768052	chrV	Evenly Distributed	252841108	119104114
346	chrV:9911653	chrV	Evenly Distributed	418642165	na
347	chrV:10028353	chrV	Evenly Distributed	418642167	na
348	chrV:10050428	chrV	Evenly Distributed	418642168	na
349	chrV:10355139	chrV	Evenly Distributed	418642170	na
350	chrV:10426342	chrV	Evenly Distributed	418642171	na
351	chrV:10470290	chrV	Evenly Distributed	418642172	na
352	chrV:10649179	chrV	Evenly Distributed	252841089	119104095
353	chrV:10674055	chrV	Evenly Distributed	418642173	na
354	chrV:10755616	chrV	Evenly Distributed	418642174	na
355	chrV:10781761	chrV	Evenly Distributed	418642175	na
356	chrV:10841019	chrV	Evenly Distributed	418642176	na
357	chrV:11289138	chrV	Evenly Distributed	244222819	119103769
358	chrV:11316476	chrV	Evenly Distributed	252841077	119104083
359	chrV:11368893	chrV	Evenly Distributed	418642177	na
360	chrV:11542501	chrV	Evenly Distributed	120258452	119103412
361	chrV:11642284	chrV	Evenly Distributed	418642179	na
362	chrV:11862139	chrV	Evenly Distributed	418642181	na
363	chrVI:218630	chrVI	Evenly Distributed	244222820	119103770
364	chrVI:487411	chrVI	Evenly Distributed	418642183	na
365	chrVI:657036	chrVI	Evenly Distributed	244222822	119103772
366	chrVI:1440771	chrVI	Evenly Distributed	244222823	119103773
367	chrVI:3116218	chrVI	Evenly Distributed	244222825	119103775
368	chrVI:5489579	chrVI	Evenly Distributed	418642185	na
369	chrVI:5777528	chrVI	Evenly Distributed	244222826	119103776
370	chrVI:6312798	chrVI	Evenly Distributed	418642187	na
371	chrVI:7249692	chrVI	Evenly Distributed	418642188	na
372	chrVI:8169471	chrVI	Evenly Distributed	244222827	119103777
373	chrVI:9116546	chrVI	Candidate	418642707	na
374	chrVI:9116673	chrVI	Candidate	418642674	na
375	chrVI:10415741	chrVI	Evenly Distributed	418641920	na
376	chrVI:11873663	chrVI	Evenly Distributed	120258454	119103414
377	chrVI:11954719	chrVI	Evenly Distributed	418642192	na
378	chrVI:12427477	chrVI	Evenly Distributed	418642193	na
379	chrVI:12537574	chrVI	Evenly Distributed	418642194	na
380	chrVI:12783021	chrVI	Evenly Distributed	418641899	na
381	chrVI:13220597	chrVI	Evenly Distributed	252841044	119104050
382	chrVI:13514193	chrVI	Evenly Distributed	252841099	119104105
383	chrVI:13682067	chrVI	Evenly Distributed	252841110	119104116
384	chrVI:13775642	chrVI	Evenly Distributed	418642196	na
385	chrVI:13911632	chrVI	Evenly Distributed	418642197	na
386	chrVI:14131973	chrVI	Evenly Distributed	252841117	119104123
387	chrVI:14390647	chrVI	Evenly Distributed	418642199	na
388	chrVI:14571427	chrVI	Evenly Distributed	418642200	na

389	chrVI:14585940	chrVI	Candidate	418642708	na
390	chrVI:14587926	chrVI	Candidate	418642675	na
391	chrVI:14976508	chrVI	Evenly Distributed	418642201	na
392	chrVI:15041940	chrVI	Evenly Distributed	244222829	119103779
393	chrVI:15274689	chrVI	Evenly Distributed	244222830	119103780
394	chrVI:15390272	chrVI	Evenly Distributed	244222831	119103781
395	chrVI:15413799	chrVI	Evenly Distributed	418642203	na
396	chrVI:15586470	chrVI	Evenly Distributed	244222832	119103782
397	chrVI:15654034	chrVI	Evenly Distributed	418642204	na
398	chrVI:15692312	chrVI	Evenly Distributed	418642205	na
399	chrVI:15780594	chrVI	Evenly Distributed	244222833	119103783
400	chrVI:16870159	chrVI	Evenly Distributed	244222834	119103784
401	chrVI:17071793	chrVI	Evenly Distributed	418642208	na
402	chrVII:245717	chrVII	Evenly Distributed	418642211	na
403	chrVII:286225	chrVII	Evenly Distributed	252841088	119104094
404	chrVII:330141	chrVII	Evenly Distributed	418642212	na
405	chrVII:393417	chrVII	Evenly Distributed	418642213	na
406	chrVII:537136	chrVII	Evenly Distributed	252841113	119104119
407	chrVII:743655	chrVII	Evenly Distributed	418642215	na
408	chrVII:835236	chrVII	Evenly Distributed	252841091	119104097
409	chrVII:1481322	chrVII	Evenly Distributed	418642217	na
410	chrVII:1521362	chrVII	Evenly Distributed	120258455	119103415
411	chrVII:1569236	chrVII	Evenly Distributed	418642218	na
412	chrVII:2559099	chrVII	Evenly Distributed	418642220	na
413	chrVII:2701531	chrVII	Evenly Distributed	418642221	na
414	chrVII:2737388	chrVII	Evenly Distributed	418642222	na
415	chrVII:3826986	chrVII	Evenly Distributed	418642224	na
416	chrVII:3967786	chrVII	Evenly Distributed	418641900	na
417	chrVII:4129303	chrVII	Evenly Distributed	418641921	na
418	chrVII:4310181	chrVII	Evenly Distributed	418642225	na
419	chrVII:4918254	chrVII	Evenly Distributed	418642226	na
420	chrVII:5151523	chrVII	Evenly Distributed	418642227	na
421	chrVII:5552972	chrVII	Evenly Distributed	252841066	119104072
422	chrVII:5936068	chrVII	Evenly Distributed	120258457	119103417
423	chrVII:13205977	chrVII	Candidate	252841062	119104068
424	chrVII:13373948	chrVII	Candidate	418642230	na
425	chrVII:13452516	chrVII	Evenly Distributed	244222836	119103786
426	chrVII:13525838	chrVII	Candidate	244222837	119103787
427	chrVII:15237354	chrVII	Candidate	252841075	119104081
428	chrVII:15423396	chrVII	Candidate	418642231	na
429	chrVII:16848769	chrVII	Evenly Distributed	418642232	na
430	chrVII:17108375	chrVII	Candidate	418642233	na
431	chrVII:17369940	chrVII	Candidate	244222838	119103788
432	chrVII:17992851	chrVII	Candidate	252841176	119104182
433	chrVII:17994452	chrVII	Candidate	252841175	119104181
434	chrVII:17995892	chrVII	Candidate	120258458	119103418
435	chrVII:17997544	chrVII	Candidate	252841166	119104172
436	chrVII:18152723	chrVII	Candidate	418642234	na
437	chrVII:18294966	chrVII	Candidate	418642235	na
438	chrVII:18353106	chrVII	Evenly Distributed	244222839	119103789
439	chrVII:18528048	chrVII	Candidate	418642236	na
440	chrVII:19857837	chrVII	Evenly Distributed	418642237	na
441	chrVII:20883742	chrVII	Evenly Distributed	252841067	119104073
442	chrVII:21302029	chrVII	Evenly Distributed	418642238	na
443	chrVII:21369768	chrVII	Candidate	418642239	na
444	chrVII:22798737	chrVII	Evenly Distributed	418642240	na

445	chrVII:22842571	chrVII	Candidate	252841120	119104126
446	chrVII:23463111	chrVII	Evenly Distributed	252841116	119104122
447	chrVII:23703797	chrVII	Evenly Distributed	418642243	na
448	chrVII:24052347	chrVII	Evenly Distributed	418642244	na
449	chrVII:24203557	chrVII	Evenly Distributed	120258459	119103419
450	chrVII:24217606	chrVII	Evenly Distributed	418642245	na
451	chrVII:24331980	chrVII	Evenly Distributed	244222840	119103790
452	chrVII:24610097	chrVII	Evenly Distributed	252841098	119104104
453	chrVII:25193081	chrVII	Evenly Distributed	418642246	na
454	chrVII:25257754	chrVII	Evenly Distributed	244222841	119103791
455	chrVII:25662266	chrVII	Evenly Distributed	120258460	119103420
456	chrVII:25910223	chrVII	Evenly Distributed	418642247	na
457	chrVII:25986275	chrVII	Evenly Distributed	418642248	na
458	chrVII:26082590	chrVII	Evenly Distributed	418642249	na
459	chrVII:26227403	chrVII	Evenly Distributed	120258461	119103421
460	chrVII:26395906	chrVII	Evenly Distributed	418642250	na
461	chrVII:26448674	chrVII	Evenly Distributed	252841125	119104131
462	chrVII:26538823	chrVII	Evenly Distributed	244222842	119103792
463	chrVII:26769148	chrVII	Evenly Distributed	418642251	na
464	chrVII:27881313	chrVII	Evenly Distributed	418642256	na
465	chrVII:27918897	chrVII	Evenly Distributed	418642257	na
466	chrVIII:868226	chrVIII	Evenly Distributed	418642258	na
467	chrVIII:1006141	chrVIII	Evenly Distributed	418642259	na
468	chrVIII:1293153	chrVIII	Evenly Distributed	252841114	119104120
469	chrVIII:1929053	chrVIII	Evenly Distributed	244222843	119103793
470	chrVIII:2257915	chrVIII	Evenly Distributed	418642261	na
471	chrVIII:2505620	chrVIII	Evenly Distributed	418642263	na
472	chrVIII:3153797	chrVIII	Candidate	418642264	na
473	chrVIII:3281178	chrVIII	Evenly Distributed	120258463	119103423
474	chrVIII:3455699	chrVIII	Evenly Distributed	252841156	119104162
475	chrVIII:3627706	chrVIII	Evenly Distributed	244222844	119103794
476	chrVIII:3765115	chrVIII	Evenly Distributed	418642265	na
477	chrVIII:3987295	chrVIII	Evenly Distributed	120258464	119103424
478	chrVIII:4058506	chrVIII	Evenly Distributed	418642266	na
479	chrVIII:4359755	chrVIII	Evenly Distributed	120258465	119103425
480	chrVIII:4503012	chrVIII	Evenly Distributed	244222845	119103795
481	chrVIII:5862371	chrVIII	Evenly Distributed	418642267	na
482	chrVIII:8032654	chrVIII	Candidate	418642270	na
483	chrVIII:8447445	chrVIII	Candidate	418642271	na
484	chrVIII:8858242	chrVIII	Assembly	418642273	na
485	chrVIII:9267165	chrVIII	Candidate	418642274	na
486	chrVIII:9763365	chrVIII	Candidate	252841173	119104179
487	chrVIII:9765765	chrVIII	Candidate	418642709	na
488	chrVIII:9768150	chrVIII	Candidate	252841162	119104168
489	chrVIII:9849962	chrVIII	Candidate	252841092	119104098
490	chrVIII:9851199	chrVIII	Evenly Distributed	418642275	na
491	chrVIII:12472630	chrVIII	Evenly Distributed	252841158	119104164
492	chrVIII:13412707	chrVIII	Evenly Distributed	244222846	119103796
493	chrVIII:13577518	chrVIII	Evenly Distributed	252841097	119104103
494	chrVIII:13613729	chrVIII	Evenly Distributed	244222847	119103797
495	chrVIII:14278829	chrVIII	Evenly Distributed	418642277	na
496	chrVIII:14441487	chrVIII	Evenly Distributed	418642278	na
497	chrVIII:14472465	chrVIII	Evenly Distributed	244222848	119103798
498	chrVIII:15261158	chrVIII	Evenly Distributed	418642279	na
499	chrVIII:15765679	chrVIII	Evenly Distributed	418642281	na
500	chrVIII:15993592	chrVIII	Evenly Distributed	244222849	119103799

501	chrVIII:16262675	chrVIII	Evenly Distributed	244222851	119103801
502	chrVIII:16268996	chrVIII	Evenly Distributed	418642282	na
503	chrVIII:16299555	chrVIII	Evenly Distributed	418642283	na
504	chrVIII:16518371	chrVIII	Evenly Distributed	418641901	na
505	chrVIII:16649103	chrVIII	Evenly Distributed	418642284	na
506	chrVIII:16826029	chrVIII	Evenly Distributed	244222852	119103802
507	chrVIII:16954373	chrVIII	Evenly Distributed	244222853	119103803
508	chrVIII:17359071	chrVIII	Evenly Distributed	252841141	119104147
509	chrVIII:17576018	chrVIII	Evenly Distributed	120258466	119103426
510	chrVIII:18047605	chrVIII	Evenly Distributed	244222854	119103804
511	chrVIII:18432598	chrVIII	Evenly Distributed	120258467	119103427
512	chrVIII:18685222	chrVIII	Evenly Distributed	120258468	119103428
513	chrVIII:18760705	chrVIII	Evenly Distributed	244222855	119103805
514	chrIX:216547	chrIX	Evenly Distributed	418642287	na
515	chrIX:330694	chrIX	Evenly Distributed	418642288	na
516	chrIX:430462	chrIX	Evenly Distributed	418642289	na
517	chrIX:591808	chrIX	Evenly Distributed	418642290	na
518	chrIX:639609	chrIX	Evenly Distributed	244222856	119103806
519	chrIX:670178	chrIX	Evenly Distributed	418642291	na
520	chrIX:803523	chrIX	Evenly Distributed	252841065	119104071
521	chrIX:1273244	chrIX	Evenly Distributed	244222857	119103807
522	chrIX:1452520	chrIX	Evenly Distributed	418642293	na
523	chrIX:1964025	chrIX	Evenly Distributed	418642297	na
524	chrIX:2089567	chrIX	Evenly Distributed	244222858	119103808
525	chrIX:2119661	chrIX	Evenly Distributed	418642298	na
526	chrIX:2251670	chrIX	Evenly Distributed	252841064	119104070
527	chrIX:2310926	chrIX	Evenly Distributed	418642299	na
528	chrIX:2360337	chrIX	Evenly Distributed	244222859	119103809
529	chrIX:2568576	chrIX	Evenly Distributed	418642300	na
530	chrIX:3019591	chrIX	Evenly Distributed	418642301	na
531	chrIX:5004198	chrIX	Evenly Distributed	418641922	na
532	chrIX:5109672	chrIX	Evenly Distributed	244222860	119103810
533	chrIX:5329484	chrIX	Evenly Distributed	244222861	119103811
534	chrIX:5403530	chrIX	Evenly Distributed	120258474	119103434
535	chrIX:5441237	chrIX	Evenly Distributed	244222862	119103812
536	chrIX:5568375	chrIX	Evenly Distributed	244222863	119103813
537	chrIX:5895159	chrIX	Evenly Distributed	418642302	na
538	chrIX:6126845	chrIX	Evenly Distributed	252841056	119104062
539	chrIX:7085382	chrIX	Evenly Distributed	418641923	na
540	chrIX:7146708	chrIX	Evenly Distributed	418642304	na
541	chrIX:7893416	chrIX	Evenly Distributed	418642306	na
542	chrIX:8217812	chrIX	Evenly Distributed	418642307	na
543	chrIX:8586014	chrIX	Candidate	244223070	119104020
544	chrIX:8594239	chrIX	Candidate	418642676	na
545	chrIX:8719760	chrIX	Candidate	120258475	119103435
546	chrIX:8851078	chrIX	Candidate	252841178	119104184
547	chrIX:8852807	chrIX	Candidate	244223071	119104021
548	chrIX:10468143	chrIX	Evenly Distributed	244222864	119103814
549	chrIX:10829183	chrIX	Evenly Distributed	418642308	na
550	chrIX:11528255	chrIX	Evenly Distributed	418642309	na
551	chrIX:12255563	chrIX	Candidate	120258476	119103436
552	chrIX:12869521	chrIX	Evenly Distributed	252841147	119104153
553	chrIX:12933483	chrIX	Evenly Distributed	244222865	119103815
554	chrIX:12982163	chrIX	Evenly Distributed	244222866	119103816
555	chrIX:13553866	chrIX	Evenly Distributed	252841127	119104133
556	chrIX:15475540	chrIX	Evenly Distributed	418642312	na

557	chrIX:15670033	chrIX	Evenly Distributed	244222868	119103818
558	chrIX:16381252	chrIX	Evenly Distributed	418642313	na
559	chrIX:16779825	chrIX	Evenly Distributed	244222869	119103819
560	chrIX:18447804	chrIX	Evenly Distributed	418642316	na
561	chrIX:18494397	chrIX	Evenly Distributed	418642317	na
562	chrIX:18826248	chrIX	Evenly Distributed	418642319	na
563	chrIX:18942598	chrIX	Evenly Distributed	120258478	119103438
564	chrIX:19322448	chrIX	Evenly Distributed	418642320	na
565	chrIX:19646525	chrIX	Evenly Distributed	418641924	na
566	chrIX:19745222	chrIX	Evenly Distributed	418642321	na
567	chrIX:19781202	chrIX	Evenly Distributed	244222870	119103820
568	chrIX:19859973	chrIX	Candidate	418642322	na
569	chrIX:20090929	chrIX	Evenly Distributed	244222871	119103821
570	chrX:225704	chrX	Evenly Distributed	418642323	na
571	chrX:251289	chrX	Evenly Distributed	418642324	na
572	chrX:1010922	chrX	Evenly Distributed	418642325	na
573	chrX:1245433	chrX	Evenly Distributed	120258479	119103439
574	chrX:1275840	chrX	Evenly Distributed	418642326	na
575	chrX:1488158	chrX	Evenly Distributed	120258480	119103440
576	chrX:1872613	chrX	Evenly Distributed	418642327	na
577	chrX:3182187	chrX	Candidate	244222872	119103822
578	chrX:3250052	chrX	Evenly Distributed	418642328	na
579	chrX:3419303	chrX	Evenly Distributed	120258481	119103441
580	chrX:4001681	chrX	Evenly Distributed	418642329	na
581	chrX:5784504	chrX	Evenly Distributed	252841138	119104144
582	chrX:7113953	chrX	Evenly Distributed	120258483	119103443
583	chrX:7790350	chrX	Evenly Distributed	120258484	119103444
584	chrX:8647016	chrX	Evenly Distributed	244222873	119103823
585	chrX:8668515	chrX	Evenly Distributed	418641925	na
586	chrX:8703061	chrX	Evenly Distributed	120258485	119103445
587	chrX:8761412	chrX	Evenly Distributed	418642334	na
588	chrX:9396721	chrX	Evenly Distributed	252841115	119104121
589	chrX:9829411	chrX	Candidate	252841153	119104159
590	chrX:9953026	chrX	Evenly Distributed	418642337	na
591	chrX:10080391	chrX	Evenly Distributed	418642338	na
592	chrX:10415917	chrX	Evenly Distributed	252841095	119104101
593	chrX:10845662	chrX	Evenly Distributed	244222874	119103824
594	chrX:11097543	chrX	Evenly Distributed	418642340	na
595	chrX:11139448	chrX	Evenly Distributed	252841128	119104134
596	chrX:11252137	chrX	Evenly Distributed	244222875	119103825
597	chrX:11317926	chrX	Evenly Distributed	244222876	119103826
598	chrX:11594157	chrX	Candidate	418642342	na
599	chrX:11660862	chrX	Candidate	418642343	na
600	chrX:11892705	chrX	Candidate	418642344	na
601	chrX:11947465	chrX	Evenly Distributed	418642345	na
602	chrX:12118532	chrX	Evenly Distributed	418642346	na
603	chrX:12315233	chrX	Evenly Distributed	418642347	na
604	chrX:12385349	chrX	Evenly Distributed	418642348	na
605	chrX:12507632	chrX	Evenly Distributed	244222877	119103827
606	chrX:12844036	chrX	Evenly Distributed	418642350	na
607	chrX:13132917	chrX	Evenly Distributed	418642352	na
608	chrX:13452742	chrX	Evenly Distributed	418642354	na
609	chrX:13618226	chrX	Evenly Distributed	418642355	na
610	chrX:13957504	chrX	Evenly Distributed	418641902	na
611	chrX:14220040	chrX	Evenly Distributed	418642357	na
612	chrX:14265366	chrX	Evenly Distributed	120258486	119103446

613	chrX:14456479	chrX	Evenly Distributed	252841100	119104106
614	chrX:14549101	chrX	Evenly Distributed	252841122	119104128
615	chrX:14831394	chrX	Evenly Distributed	418642358	na
616	chrX:15290785	chrX	Evenly Distributed	252841137	119104143
617	chrXI:234849	chrXI	Evenly Distributed	120258487	119103447
618	chrXI:457909	chrXI	Evenly Distributed	244222878	119103828
619	chrXI:965263	chrXI	Assembly	244222879	119103829
620	chrXI:1266618	chrXI	Assembly	418642362	na
621	chrXI:1333427	chrXI	Candidate	418642363	na
622	chrXI:1449684	chrXI	Evenly Distributed	120258489	119103449
623	chrXI:1680578	chrXI	Evenly Distributed	418642364	na
624	chrXI:1921360	chrXI	Evenly Distributed	120258490	119103450
625	chrXI:2771408	chrXI	Evenly Distributed	418642365	na
626	chrXI:3120961	chrXI	Evenly Distributed	244222880	119103830
627	chrXI:3902043	chrXI	Candidate	418642367	na
628	chrXI:5254527	chrXI	Candidate	418642368	na
629	chrXI:5472842	chrXI	Candidate	244223076	119104026
630	chrXI:5472952	chrXI	Candidate	418642677	na
631	chrXI:5550667	chrXI	Candidate	418642710	na
632	chrXI:5652180	chrXI	Candidate	244223077	119104027
633	chrXI:5708414	chrXI	Candidate	244222881	119103831
634	chrXI:5752298	chrXI	Candidate	244223080	119104030
635	chrXI:5845597	chrXI	Candidate	244223081	119104031
636	chrXI:5845760	chrXI	Candidate	252841177	119104183
637	chrXI:7355052	chrXI	Evenly Distributed	418642370	na
638	chrXI:7370453	chrXI	Candidate	418642371	na
639	chrXI:7381607	chrXI	Candidate	418642372	na
640	chrXI:7635920	chrXI	Candidate	418642678	na
641	chrXI:9039275	chrXI	Evenly Distributed	252841094	119104100
642	chrXI:10442904	chrXI	Evenly Distributed	244222882	119103832
643	chrXI:10717955	chrXI	Candidate	418642373	na
644	chrXI:10819967	chrXI	Candidate	418642374	na
645	chrXI:10976029	chrXI	Evenly Distributed	244222883	119103833
646	chrXI:12097498	chrXI	Evenly Distributed	418642375	na
647	chrXI:12550151	chrXI	Evenly Distributed	418642376	na
648	chrXI:12746496	chrXI	Evenly Distributed	244222884	119103834
649	chrXI:14286902	chrXI	Evenly Distributed	120258493	119103453
650	chrXI:14426451	chrXI	Evenly Distributed	418642377	na
651	chrXI:14541455	chrXI	Evenly Distributed	418642378	na
652	chrXI:14616764	chrXI	Evenly Distributed	120258494	119103454
653	chrXI:14691162	chrXI	Evenly Distributed	418642380	na
654	chrXI:14830913	chrXI	Evenly Distributed	244222885	119103835
655	chrXI:14877858	chrXI	Evenly Distributed	418642381	na
656	chrXI:15005173	chrXI	Evenly Distributed	244222886	119103836
657	chrXI:15154801	chrXI	Evenly Distributed	418642382	na
658	chrXI:15600885	chrXI	Evenly Distributed	244222887	119103837
659	chrXI:15962189	chrXI	Evenly Distributed	418642383	na
660	chrXI:16451866	chrXI	Evenly Distributed	418642384	na
661	chrXI:16494449	chrXI	Evenly Distributed	418642385	na
662	chrXI:16655205	chrXI	Evenly Distributed	120258495	119103455
663	chrXI:16701186	chrXI	Evenly Distributed	244222888	119103838
664	chrXII:70478	chrXII	Evenly Distributed	418642386	na
665	chrXII:548804	chrXII	Evenly Distributed	252841119	119104125
666	chrXII:699742	chrXII	Evenly Distributed	418642387	na
667	chrXII:857247	chrXII	Evenly Distributed	120258496	119103456
668	chrXII:880748	chrXII	Evenly Distributed	418642389	na

669	chrXII:1483544	chrXII	Evenly Distributed	244222889	119103839
670	chrXII:1657077	chrXII	Evenly Distributed	418642391	na
671	chrXII:1969537	chrXII	Evenly Distributed	252841070	119104076
672	chrXII:2034315	chrXII	Evenly Distributed	418642392	na
673	chrXII:2157795	chrXII	Evenly Distributed	418642393	na
674	chrXII:2181073	chrXII	Evenly Distributed	252841129	119104135
675	chrXII:2242677	chrXII	Evenly Distributed	418642394	na
676	chrXII:2615696	chrXII	Evenly Distributed	418642396	na
677	chrXII:2713984	chrXII	Evenly Distributed	418642397	na
678	chrXII:3026329	chrXII	Evenly Distributed	418642398	na
679	chrXII:3810254	chrXII	Evenly Distributed	418642399	na
680	chrXII:3894122	chrXII	Evenly Distributed	244222890	119103840
681	chrXII:4042442	chrXII	Evenly Distributed	244222891	119103841
682	chrXII:4123972	chrXII	Evenly Distributed	418642400	na
683	chrXII:4550244	chrXII	Evenly Distributed	418641926	na
684	chrXII:5828898	chrXII	Evenly Distributed	418642403	na
685	chrXII:6012527	chrXII	Evenly Distributed	418642404	na
686	chrXII:6399147	chrXII	Evenly Distributed	252841133	119104139
687	chrXII:6745006	chrXII	Evenly Distributed	244222892	119103842
688	chrXII:6913126	chrXII	Evenly Distributed	120258500	119103460
689	chrXII:6924609	chrXII	Evenly Distributed	418642405	na
690	chrXII:7504339	chrXII	Evenly Distributed	418642406	na
691	chrXII:8640908	chrXII	Evenly Distributed	120258501	119103461
692	chrXII:10243906	chrXII	Candidate	252841053	119104059
693	chrXII:10246245	chrXII	Candidate	252841052	119104058
694	chrXII:11472159	chrXII	Evenly Distributed	418642407	na
695	chrXII:12327199	chrXII	Candidate	418642408	na
696	chrXII:12773355	chrXII	Candidate	418642409	na
697	chrXII:13004645	chrXII	Candidate	244222893	119103843
698	chrXII:13045611	chrXII	Evenly Distributed	244222894	119103844
699	chrXII:13151755	chrXII	Candidate	244223082	119104032
700	chrXII:14223760	chrXII	Evenly Distributed	244222895	119103845
701	chrXII:14344087	chrXII	Candidate	418642711	na
702	chrXII:14346080	chrXII	Candidate	244223084	119104034
703	chrXII:14353450	chrXII	Candidate	244222896	119103846
704	chrXII:15046849	chrXII	Evenly Distributed	418642410	na
705	chrXII:16454328	chrXII	Evenly Distributed	418642411	na
706	chrXII:16628544	chrXII	Evenly Distributed	418642412	na
707	chrXII:17758877	chrXII	Evenly Distributed	244222897	119103847
708	chrXII:18091460	chrXII	Evenly Distributed	418642417	na
709	chrXII:18196420	chrXII	Evenly Distributed	244222898	119103848
710	chrXII:18221941	chrXII	Evenly Distributed	244222899	119103849
711	chrXIII:36455	chrXIII	Candidate	418642712	na
712	chrXIII:531198	chrXIII	Evenly Distributed	418642419	na
713	chrXIII:1001571	chrXIII	Evenly Distributed	120258503	119103463
714	chrXIII:1388673	chrXIII	Evenly Distributed	120258504	119103464
715	chrXIII:1698554	chrXIII	Evenly Distributed	418642421	na
716	chrXIII:1909687	chrXIII	Evenly Distributed	244222900	119103850
717	chrXIII:2105469	chrXIII	Evenly Distributed	418642423	na
718	chrXIII:2523163	chrXIII	Evenly Distributed	120258505	119103465
719	chrXIII:2632698	chrXIII	Evenly Distributed	244222901	119103851
720	chrXIII:2822352	chrXIII	Evenly Distributed	244222902	119103852
721	chrXIII:2969182	chrXIII	Evenly Distributed	418642424	na
722	chrXIII:4401535	chrXIII	Evenly Distributed	418642425	na
723	chrXIII:4621027	chrXIII	Evenly Distributed	418642426	na
724	chrXIII:4868788	chrXIII	Evenly Distributed	418642428	na

725	chrXIII:5772867	chrXIII	Evenly Distributed	418642429	na
726	chrXIII:7266499	chrXIII	Evenly Distributed	120258508	119103468
727	chrXIII:8085851	chrXIII	Evenly Distributed	418642430	na
728	chrXIII:12083700	chrXIII	Candidate	252841126	119104132
729	chrXIII:14401483	chrXIII	Candidate	252841160	119104166
730	chrXIII:14403124	chrXIII	Candidate	418642679	na
731	chrXIII:15462116	chrXIII	Evenly Distributed	244222903	119103853
732	chrXIII:15799426	chrXIII	Evenly Distributed	418642431	na
733	chrXIII:17052511	chrXIII	Evenly Distributed	244222904	119103854
734	chrXIII:17249562	chrXIII	Evenly Distributed	418642432	na
735	chrXIII:17392141	chrXIII	Evenly Distributed	120258510	119103470
736	chrXIII:17896505	chrXIII	Evenly Distributed	252841131	119104137
737	chrXIII:18043261	chrXIII	Evenly Distributed	244222905	119103855
738	chrXIII:18470329	chrXIII	Evenly Distributed	252841124	119104130
739	chrXIII:19311265	chrXIII	Evenly Distributed	252841123	119104129
740	chrXIII:19318277	chrXIII	Candidate	418642680	na
741	chrXIII:19693259	chrXIII	Evenly Distributed	252841080	119104086
742	chrXIII:19783975	chrXIII	Candidate	418642681	na
743	chrXIII:19824333	chrXIII	Evenly Distributed	244222906	119103856
744	chrXIII:20036656	chrXIII	Assembly	418642433	na
745	chrXIII:20057491	chrXIII	Candidate	418642713	na
746	chrXIV:55971	chrXIV	Evenly Distributed	418641903	na
747	chrXIV:97123	chrXIV	Evenly Distributed	418642434	na
748	chrXIV:265589	chrXIV	Evenly Distributed	252841057	119104063
749	chrXIV:451065	chrXIV	Evenly Distributed	120258511	119103471
750	chrXIV:628299	chrXIV	Evenly Distributed	418642437	na
751	chrXIV:721170	chrXIV	Evenly Distributed	244222907	119103857
752	chrXIV:800076	chrXIV	Evenly Distributed	418642438	na
753	chrXIV:1087388	chrXIV	Evenly Distributed	418642439	na
754	chrXIV:1220813	chrXIV	Evenly Distributed	418642440	na
755	chrXIV:1311694	chrXIV	Evenly Distributed	418642441	na
756	chrXIV:1383447	chrXIV	Evenly Distributed	244222908	119103858
757	chrXIV:1442872	chrXIV	Evenly Distributed	120258512	119103472
758	chrXIV:1641269	chrXIV	Evenly Distributed	418642442	na
759	chrXIV:1713227	chrXIV	Evenly Distributed	120258513	119103473
760	chrXIV:1798136	chrXIV	Evenly Distributed	418642443	na
761	chrXIV:1871946	chrXIV	Candidate	418642714	na
762	chrXIV:2084777	chrXIV	Evenly Distributed	418642446	na
763	chrXIV:2165223	chrXIV	Evenly Distributed	418642447	na
764	chrXIV:2183258	chrXIV	Evenly Distributed	418642448	na
765	chrXIV:2281005	chrXIV	Evenly Distributed	418641927	na
766	chrXIV:2345947	chrXIV	Evenly Distributed	244222909	119103859
767	chrXIV:2908977	chrXIV	Evenly Distributed	418642451	na
768	chrXIV:3414352	chrXIV	Evenly Distributed	120258514	119103474
769	chrXIV:3534175	chrXIV	Evenly Distributed	120258515	119103475
770	chrXIV:3598443	chrXIV	Evenly Distributed	418642452	na
771	chrXIV:3690040	chrXIV	Evenly Distributed	418642453	na
772	chrXIV:3914017	chrXIV	Evenly Distributed	120258516	119103476
773	chrXIV:4632223	chrXIV	Evenly Distributed	418642454	na
774	chrXIV:6641188	chrXIV	Evenly Distributed	418642455	na
775	chrXIV:9742642	chrXIV	Evenly Distributed	418642458	na
776	chrXIV:10399121	chrXIV	Evenly Distributed	244222910	119103860
777	chrXIV:10781747	chrXIV	Evenly Distributed	418642459	na
778	chrXIV:11054767	chrXIV	Evenly Distributed	120258517	119103477
779	chrXIV:11360680	chrXIV	Evenly Distributed	252841142	119104148
780	chrXIV:14049917	chrXIV	Evenly Distributed	252841090	119104096

781	chrXIV:15033103	chrXIV	Evenly Distributed	418642461	na
782	chrXIV:15137805	chrXIV	Evenly Distributed	418642462	na
783	chrXV:11818	chrXV	Assembly	418642463	na
784	chrXV:137728	chrXV	Evenly Distributed	120258518	119103478
785	chrXV:414608	chrXV	Evenly Distributed	120258519	119103479
786	chrXV:979445	chrXV	Evenly Distributed	418642466	na
787	chrXV:1027745	chrXV	Candidate	418642715	na
788	chrXV:1029767	chrXV	Candidate	244223088	119104038
789	chrXV:1354426	chrXV	Evenly Distributed	418642467	na
790	chrXV:1800560	chrXV	Evenly Distributed	418642468	na
791	chrXV:1902350	chrXV	Evenly Distributed	244222911	119103861
792	chrXV:2169610	chrXV	Evenly Distributed	244222912	119103862
793	chrXV:2507809	chrXV	Evenly Distributed	244222914	119103864
794	chrXV:2845394	chrXV	Candidate	418642472	na
795	chrXV:2964371	chrXV	Evenly Distributed	418641928	na
796	chrXV:3237255	chrXV	Evenly Distributed	418642473	na
797	chrXV:3568577	chrXV	Evenly Distributed	244222915	119103865
798	chrXV:3703641	chrXV	Evenly Distributed	418642475	na
799	chrXV:3871813	chrXV	Evenly Distributed	418642476	na
800	chrXV:5929959	chrXV	Candidate	244222916	119103866
801	chrXV:6446874	chrXV	Evenly Distributed	418642477	na
802	chrXV:7200442	chrXV	Candidate	244223089	119104039
803	chrXV:10155822	chrXV	Evenly Distributed	418642478	na
804	chrXV:12281774	chrXV	Evenly Distributed	418642480	na
805	chrXV:13047331	chrXV	Evenly Distributed	418642481	na
806	chrXV:14693722	chrXV	Evenly Distributed	244222917	119103867
807	chrXV:15139567	chrXV	Evenly Distributed	244222918	119103868
808	chrXV:15732540	chrXV	Evenly Distributed	418641904	na
809	chrXV:15780243	chrXV	Evenly Distributed	418642482	na
810	chrXV:15848836	chrXV	Evenly Distributed	418642483	na
811	chrXV:16003465	chrXV	Evenly Distributed	244222919	119103869
812	chrXV:16193822	chrXV	Evenly Distributed	244222920	119103870
813	chrXVI:325117	chrXVI	Evenly Distributed	418642484	na
814	chrXVI:427091	chrXVI	Evenly Distributed	418641905	na
815	chrXVI:1267322	chrXVI	Evenly Distributed	418642485	na
816	chrXVI:2068005	chrXVI	Evenly Distributed	120258522	119103482
817	chrXVI:2392758	chrXVI	Evenly Distributed	244222921	119103871
818	chrXVI:2483136	chrXVI	Evenly Distributed	252841051	119104057
819	chrXVI:2650854	chrXVI	Evenly Distributed	244222922	119103872
820	chrXVI:2764206	chrXVI	Evenly Distributed	120258523	119103483
821	chrXVI:3206769	chrXVI	Evenly Distributed	244222923	119103873
822	chrXVI:3719447	chrXVI	Evenly Distributed	418641906	na
823	chrXVI:4906748	chrXVI	Evenly Distributed	418642486	na
824	chrXVI:5562355	chrXVI	Evenly Distributed	244222924	119103874
825	chrXVI:6415385	chrXVI	Evenly Distributed	418642487	na
826	chrXVI:6451650	chrXVI	Candidate	244222925	119103875
827	chrXVI:8218481	chrXVI	Evenly Distributed	418642488	na
828	chrXVI:9428786	chrXVI	Evenly Distributed	244222926	119103876
829	chrXVI:9857539	chrXVI	Candidate	244222927	119103877
830	chrXVI:9981125	chrXVI	Evenly Distributed	244222928	119103878
831	chrXVI:11214322	chrXVI	Evenly Distributed	418642489	na
832	chrXVI:11738218	chrXVI	Evenly Distributed	418642490	na
833	chrXVI:12111717	chrXVI	Evenly Distributed	120258526	119103486
834	chrXVI:12128738	chrXVI	Candidate	418642491	na
835	chrXVI:12996432	chrXVI	Evenly Distributed	244222929	119103879
836	chrXVI:13148331	chrXVI	Evenly Distributed	418642492	na

837	chrXVI:13921749	chrXVI	Evenly Distributed	120258527	119103487
838	chrXVI:14093156	chrXVI	Evenly Distributed	244222931	119103881
839	chrXVI:14283264	chrXVI	Evenly Distributed	244222932	119103882
840	chrXVI:14550048	chrXVI	Evenly Distributed	252841073	119104079
841	chrXVI:14620556	chrXVI	Evenly Distributed	418642493	na
842	chrXVI:14688989	chrXVI	Evenly Distributed	252841047	119104053
843	chrXVI:14963879	chrXVI	Evenly Distributed	244222933	119103883
844	chrXVI:15039503	chrXVI	Evenly Distributed	418642494	na
845	chrXVI:15214733	chrXVI	Evenly Distributed	418641929	na
846	chrXVI:15413858	chrXVI	Evenly Distributed	418641907	na
847	chrXVI:15457430	chrXVI	Evenly Distributed	244222934	119103884
848	chrXVI:15826700	chrXVI	Evenly Distributed	244222935	119103885
849	chrXVI:15887018	chrXVI	Evenly Distributed	418642495	na
850	chrXVI:16058672	chrXVI	Evenly Distributed	252841101	119104107
851	chrXVI:16122163	chrXVI	Evenly Distributed	418642496	na
852	chrXVI:16673569	chrXVI	Evenly Distributed	120258528	119103488
853	chrXVI:16824588	chrXVI	Evenly Distributed	418642500	na
854	chrXVI:17236926	chrXVI	Evenly Distributed	244222936	119103886
855	chrXVI:17347559	chrXVI	Candidate	244223091	119104041
856	chrXVI:17405918	chrXVI	Evenly Distributed	418642501	na
857	chrXVI:17471373	chrXVI	Evenly Distributed	418642502	na
858	chrXVI:17506151	chrXVI	Assembly	252841081	119104087
859	chrXVI:17518534	chrXVI	Evenly Distributed	418642503	na
860	chrXVI:17773420	chrXVI	Evenly Distributed	244222937	119103887
861	chrXVI:17895677	chrXVI	Evenly Distributed	244222938	119103888
862	chrXVI:18106789	chrXVI	Evenly Distributed	120258529	119103489
863	chrXVII:319223	chrXVII	Evenly Distributed	418642504	na
864	chrXVII:486497	chrXVII	Evenly Distributed	418642505	na
865	chrXVII:645029	chrXVII	Assembly	418642506	na
866	chrXVII:769372	chrXVII	Assembly	244222939	119103889
867	chrXVII:950518	chrXVII	Candidate	120258531	119103491
868	chrXVII:1264852	chrXVII	Evenly Distributed	418642508	na
869	chrXVII:1733515	chrXVII	Evenly Distributed	418642509	na
870	chrXVII:2232080	chrXVII	Evenly Distributed	120258532	119103492
871	chrXVII:2626658	chrXVII	Evenly Distributed	418642511	na
872	chrXVII:2872553	chrXVII	Evenly Distributed	418642512	na
873	chrXVII:3094026	chrXVII	Evenly Distributed	244222941	119103891
874	chrXVII:3496867	chrXVII	Candidate	418642514	na
875	chrXVII:3535017	chrXVII	Evenly Distributed	418642515	na
876	chrXVII:3706521	chrXVII	Evenly Distributed	418642516	na
877	chrXVII:3843835	chrXVII	Evenly Distributed	120258534	119103494
878	chrXVII:3906379	chrXVII	Evenly Distributed	244222942	119103892
879	chrXVII:4089045	chrXVII	Evenly Distributed	418642517	na
880	chrXVII:4260411	chrXVII	Evenly Distributed	418642518	na
881	chrXVII:4793150	chrXVII	Evenly Distributed	418642519	na
882	chrXVII:4846889	chrXVII	Evenly Distributed	244222943	119103893
883	chrXVII:4909843	chrXVII	Evenly Distributed	244222944	119103894
884	chrXVII:6590666	chrXVII	Candidate	418642682	na
885	chrXVII:8770036	chrXVII	Evenly Distributed	244222945	119103895
886	chrXVII:9024413	chrXVII	Evenly Distributed	244222946	119103896
887	chrXVII:9697366	chrXVII	Evenly Distributed	244222947	119103897
888	chrXVII:9881295	chrXVII	Evenly Distributed	418642523	na
889	chrXVII:10176745	chrXVII	Evenly Distributed	244222948	119103898
890	chrXVII:10329401	chrXVII	Evenly Distributed	418642524	na
891	chrXVII:11037958	chrXVII	Evenly Distributed	252841072	119104078
892	chrXVII:11662207	chrXVII	Candidate	418642525	na

893	chrXVII:11855617	chrXVII	Evenly Distributed	120258535	119103495
894	chrXVII:12022612	chrXVII	Evenly Distributed	120258536	119103496
895	chrXVII:12528572	chrXVII	Evenly Distributed	252841151	119104157
896	chrXVII:12599208	chrXVII	Evenly Distributed	252841154	119104160
897	chrXVII:12666712	chrXVII	Evenly Distributed	418642526	na
898	chrXVII:13079654	chrXVII	Evenly Distributed	252841130	119104136
899	chrXVII:13481178	chrXVII	Evenly Distributed	120258537	119103497
900	chrXVII:13795831	chrXVII	Assembly	252841087	119104093
901	chrXVII:14127979	chrXVII	Evenly Distributed	418642528	na
902	chrXVII:14583681	chrXVII	Evenly Distributed	120258538	119103498
903	chrXVIII:1211531	chrXVIII	Evenly Distributed	418642530	na
904	chrXVIII:2251951	chrXVIII	Evenly Distributed	244222950	119103900
905	chrXVIII:2713659	chrXVIII	Evenly Distributed	418642532	na
906	chrXVIII:2915712	chrXVIII	Evenly Distributed	418642533	na
907	chrXVIII:3037920	chrXVIII	Evenly Distributed	418642534	na
908	chrXVIII:4836241	chrXVIII	Evenly Distributed	120258539	119103499
909	chrXVIII:5765162	chrXVIII	Evenly Distributed	120258540	119103500
910	chrXVIII:7142583	chrXVIII	Evenly Distributed	418642536	na
911	chrXVIII:8258269	chrXVIII	Candidate	418642537	na
912	chrXVIII:9955470	chrXVIII	Evenly Distributed	244222951	119103901
913	chrXVIII:10327897	chrXVIII	Evenly Distributed	418641930	na
914	chrXVIII:10943853	chrXVIII	Evenly Distributed	418642539	na
915	chrXVIII:11086837	chrXVIII	Evenly Distributed	120258542	119103502
916	chrXVIII:11229958	chrXVIII	Evenly Distributed	418642540	na
917	chrXVIII:11402209	chrXVIII	Evenly Distributed	244222952	119103902
918	chrXVIII:11504306	chrXVIII	Evenly Distributed	418642542	na
919	chrXVIII:11641450	chrXVIII	Evenly Distributed	244222953	119103903
920	chrXVIII:11702469	chrXVIII	Evenly Distributed	418642543	na
921	chrXVIII:11765327	chrXVIII	Evenly Distributed	120258543	119103503
922	chrXVIII:11896010	chrXVIII	Evenly Distributed	244222954	119103904
923	chrXVIII:12146976	chrXVIII	Evenly Distributed	244222955	119103905
924	chrXVIII:12273872	chrXVIII	Assembly	252841150	119104156
925	chrXVIII:12501504	chrXVIII	Evenly Distributed	244222956	119103906
926	chrXVIII:12818939	chrXVIII	Evenly Distributed	120258545	119103505
927	chrXVIII:13193140	chrXVIII	Evenly Distributed	244222957	119103907
928	chrXVIII:13352631	chrXVIII	Evenly Distributed	120258546	119103506
929	chrXVIII:13753579	chrXVIII	Evenly Distributed	244222958	119103908
930	chrXVIII:13773116	chrXVIII	Evenly Distributed	418642545	na
931	chrXVIII:14022132	chrXVIII	Evenly Distributed	418642546	na
932	chrXVIII:14076408	chrXVIII	Evenly Distributed	244222959	119103909
933	chrXVIII:14160764	chrXVIII	Evenly Distributed	418642547	na
934	chrXVIII:14415132	chrXVIII	Evenly Distributed	120258548	119103508
935	chrXVIII:15478444	chrXVIII	Evenly Distributed	120258549	119103509
936	chrXIX:377856	chrXIX	Evenly Distributed	418641954	na
937	chrXIX:583166	chrXIX	Evenly Distributed	252841105	119104111
938	chrXIX:646137	chrXIX	Evenly Distributed	252841146	119104152
939	chrXIX:707167	chrXIX	Evenly Distributed	418641955	na
940	chrXIX:728155	chrXIX	Evenly Distributed	120258550	119103510
941	chrXIX:897343	chrXIX	Evenly Distributed	418641956	na
942	chrXIX:910003	chrXIX	Evenly Distributed	244222960	119103910
943	chrXIX:1055721	chrXIX	Evenly Distributed	418641931	na
944	chrXIX:1472847	chrXIX	Evenly Distributed	120258551	119103511
945	chrXIX:1546489	chrXIX	Evenly Distributed	418641958	na
946	chrXIX:1578018	chrXIX	Evenly Distributed	418641959	na
947	chrXIX:1670068	chrXIX	Evenly Distributed	418641960	na
948	chrXIX:1706115	chrXIX	Evenly Distributed	418641961	na

949	chrXIX:1787668	chrXIX	Evenly Distributed	418641962	na
950	chrXIX:2459466	chrXIX	Candidate	244235762	119104047
951	chrXIX:2464070	chrXIX	Candidate	244235760	119104045
952	chrXIX:2467932	chrXIX	Candidate	244235759	119104044
953	chrXIX:2477173	chrXIX	Candidate	244235761	119104046
954	chrXIX:2507823	chrXIX	Candidate	418642683	na
955	chrXIX:2524656	chrXIX	Candidate	418642716	na
956	chrXIX:2524840	chrXIX	Candidate	252841167	119104173
957	chrXIX:2526286	chrXIX	Candidate	244223087	119104037
958	chrXIX:2545418	chrXIX	Candidate	418642684	na
959	chrXIX:2547581	chrXIX	Candidate	418642717	na
960	chrXIX:2547872	chrXIX	Candidate	418642685	na
961	chrXIX:2550417	chrXIX	Candidate	252841164	119104170
962	chrXIX:2854564	chrXIX	Evenly Distributed	418641963	na
963	chrXIX:3309372	chrXIX	Evenly Distributed	120258553	119103513
964	chrXIX:3737235	chrXIX	Evenly Distributed	418641965	na
965	chrXIX:5674894	chrXIX	Evenly Distributed	418641967	na
966	chrXIX:7049551	chrXIX	Candidate	418641969	na
967	chrXIX:7129928	chrXIX	Evenly Distributed	244235763	119104048
968	chrXIX:7756941	chrXIX	Candidate	418641908	na
969	chrXIX:7848104	chrXIX	Evenly Distributed	252841069	119104075
970	chrXIX:8190806	chrXIX	Evenly Distributed	120258554	119103514
971	chrXIX:9639539	chrXIX	Evenly Distributed	120258555	119103515
972	chrXIX:10552047	chrXIX	Candidate	252841043	119104049
973	chrXIX:11573473	chrXIX	Evenly Distributed	418641972	na
974	chrXIX:12707451	chrXIX	Candidate	418641909	na
975	chrXIX:13466609	chrXIX	Candidate	244222961	119103911
976	chrXIX:14575706	chrXIX	Candidate	418641974	na
977	chrXIX:14650559	chrXIX	Evenly Distributed	418641975	na
978	chrXIX:14796728	chrXIX	Candidate	244223085	119104035
979	chrXIX:14798132	chrXIX	Candidate	244223086	119104036
980	chrXIX:14799088	chrXIX	Candidate	252841180	119104186
981	chrXIX:14800062	chrXIX	Candidate	418642718	na
982	chrXIX:15207098	chrXIX	Evenly Distributed	252841144	119104150
983	chrXIX:18043409	chrXIX	Evenly Distributed	252841059	119104065
984	chrXIX:18045399	chrXIX	Evenly Distributed	120258558	119103518
985	chrXX:232763	chrXX	Candidate	244223092	119104042
986	chrXX:832599	chrXX	Evenly Distributed	418641910	na
987	chrXX:1758783	chrXX	Evenly Distributed	244222962	119103912
988	chrXX:1808773	chrXX	Evenly Distributed	418642552	na
989	chrXX:2312273	chrXX	Evenly Distributed	418642554	na
990	chrXX:2885078	chrXX	Evenly Distributed	418642555	na
991	chrXX:3608355	chrXX	Candidate	244223093	119104043
992	chrXX:3854201	chrXX	Evenly Distributed	418641911	na
993	chrXX:4435386	chrXX	Evenly Distributed	418642557	na
994	chrXX:5734841	chrXX	Evenly Distributed	418642558	na
995	chrXX:7066321	chrXX	Evenly Distributed	418642560	na
996	chrXX:8905625	chrXX	Candidate	244222963	119103913
997	chrXX:8918466	chrXX	Candidate	244222964	119103914
998	chrXX:9012094	chrXX	Assembly	418642561	na
999	chrXX:9279241	chrXX	Evenly Distributed	244222965	119103915
1000	chrXX:9974519	chrXX	Candidate	418642563	na
1001	chrXX:11525990	chrXX	Candidate	418642564	na
1002	chrXX:12436776	chrXX	Candidate	252841049	119104055
1003	chrXX:12607435	chrXX	Candidate	418642565	na
1004	chrXX:12622695	chrXX	Assembly	244222966	119103916

1005	chrXX:12681526	chrXX	Candidate	244222967	119103917
1006	chrXX:12810044	chrXX	Assembly	252841048	119104054
1007	chrXX:13893619	chrXX	Evenly Distributed	252841139	119104145
1008	chrXX:14324175	chrXX	Evenly Distributed	120258560	119103520
1009	chrXX:14411783	chrXX	Candidate	252841159	119104165
1010	chrXX:14412604	chrXX	Candidate	418642686	na
1011	chrXX:14462157	chrXX	Evenly Distributed	244222968	119103918
1012	chrXX:14859034	chrXX	Evenly Distributed	418642571	na
1013	chrXX:15425227	chrXX	Evenly Distributed	418641932	na
1014	chrXX:15784121	chrXX	Evenly Distributed	418641912	na
1015	chrXX:15996390	chrXX	Evenly Distributed	418642573	na
1016	chrXX:16111163	chrXX	Evenly Distributed	418642574	na
1017	chrXX:16137862	chrXX	Evenly Distributed	244222969	119103919
1018	chrXX:16213434	chrXX	Evenly Distributed	418642575	na
1019	chrXX:16253512	chrXX	Evenly Distributed	252841060	119104066
1020	chrXX:16328884	chrXX	Evenly Distributed	120258561	119103521
1021	chrXX:16671291	chrXX	Evenly Distributed	418641933	na
1022	chrXX:16912820	chrXX	Candidate	252841161	119104167
1023	chrXX:17494242	chrXX	Evenly Distributed	252841140	119104146
1024	chrXX:17636436	chrXX	Evenly Distributed	418642578	na
1025	chrXX:17708486	chrXX	Evenly Distributed	244222970	119103920
1026	chrXX:17770802	chrXX	Evenly Distributed	244222971	119103921
1027	chrXX:18193281	chrXX	Evenly Distributed	418642579	na
1028	chrXX:18469330	chrXX	Evenly Distributed	418642580	na
1029	chrXX:18810026	chrXX	Evenly Distributed	418641934	na
1030	chrXX:18893200	chrXX	Evenly Distributed	418641913	na
1031	chrXX:19020370	chrXX	Evenly Distributed	244222972	119103922
1032	chrXX:19066707	chrXX	Evenly Distributed	418642582	na
1033	chrXXI:242380	chrXXI	Evenly Distributed	418641935	na
1034	chrXXI:772528	chrXXI	Candidate	418642687	na
1035	chrXXI:774193	chrXXI	Candidate	252841169	119104175
1036	chrXXI:1146721	chrXXI	Evenly Distributed	418642583	na
1037	chrXXI:1893294	chrXXI	Candidate	252841157	119104163
1038	chrXXI:1895965	chrXXI	Candidate	418642584	na
1039	chrXXI:3082227	chrXXI	Evenly Distributed	418642585	na
1040	chrXXI:5648650	chrXXI	Candidate	418642688	na
1041	chrXXI:5716516	chrXXI	Candidate	252841174	119104180
1042	chrXXI:5717549	chrXXI	Candidate	418642689	na
1043	chrXXI:5737465	chrXXI	Evenly Distributed	244222973	119103923
1044	chrXXI:5791519	chrXXI	Candidate	244222974	119103924
1045	chrXXI:5793103	chrXXI	Candidate	244222975	119103925
1046	chrXXI:6037992	chrXXI	Candidate	244222976	119103926
1047	chrXXI:6356754	chrXXI	Evenly Distributed	418642586	na
1048	chrXXI:7002178	chrXXI	Assembly	244222977	119103927
1049	chrXXI:7466744	chrXXI	Candidate	418642587	na
1050	chrXXI:7544041	chrXXI	Candidate	244222978	119103928
1051	chrXXI:7904439	chrXXI	Candidate	252841107	119104113
1052	chrXXI:8268451	chrXXI	Evenly Distributed	120258563	119103523
1053	chrXXI:9373717	chrXXI	Evenly Distributed	418642588	na
1054	chrXXI:9820534	chrXXI	Evenly Distributed	418642589	na
1055	chrXXI:10007883	chrXXI	Evenly Distributed	120258564	119103524
1056	chrXXI:10054378	chrXXI	Assembly	244222979	119103929
1057	chrXXI:10156751	chrXXI	Evenly Distributed	252841143	119104149
1058	chrXXI:10236129	chrXXI	Evenly Distributed	244222980	119103930
1059	chrXXI:10969152	chrXXI	Evenly Distributed	244222981	119103931
1060	chrXXI:11060209	chrXXI	Evenly Distributed	120258566	119103526

1061	chrXXI:11179443	chrXXI	Evenly Distributed	244222982	119103932
1062	chrXXI:11414383	chrXXI	Evenly Distributed	120258567	119103527
1063	chrXXI:11532913	chrXXI	Evenly Distributed	418642590	na
1064	chrUn:498491	chrUn	Assembly	244222983	119103933
1065	chrUn:5488093	chrUn	Assembly	244222984	119103934
1066	chrUn:10646840	chrUn	Assembly	418642592	na
1067	chrUn:11621796	chrUn	Assembly	244222985	119103935
1068	chrUn:14031310	chrUn	Assembly	418642593	na
1069	chrUn:15052344	chrUn	Assembly	418642594	na
1070	chrUn:17430501	chrUn	Candidate	418642690	na
1071	chrUn:21370233	chrUn	Candidate	418642719	na
1072	chrUn:21370836	chrUn	Candidate	418642691	na
1073	chrUn:23443588	chrUn	Candidate	418642720	na
1074	chrUn:25489046	chrUn	Assembly	418642595	na
1075	chrUn:28091692	chrUn	Candidate	244223072	119104022
1076	chrUn:28539586	chrUn	Candidate	418642692	na
1077	chrUn:31468769	chrUn	Assembly	418642597	na
1078	chrUn:31913084	chrUn	Assembly	244222986	119103936
1079	chrUn:33574989	chrUn	Candidate	418642721	na
1080	chrUn:35375609	chrUn	Assembly	418642599	na
1081	chrUn:59015362	chrUn	Candidate	418642722	na
1082	chrM:827	chrM	Assembly	418641938	na
1083	chrM:2379	chrM	Assembly	418641936	na
1084	chrM:8198	chrM	Assembly	418641939	na
1085	chrM:8228	chrM	Assembly	418641940	na
1086	chrM:8965	chrM	Candidate	418641941	na
1087	chrM:14722	chrM	Assembly	418641944	na
1088	chrM:14743	chrM	Assembly	418641914	na
1089	chrM:14785	chrM	Assembly	418641945	na
1090	chrM:14803	chrM	Assembly	418641946	na
1091	chrM:14935	chrM	Assembly	418641937	na
1092	chrM:15061	chrM	Assembly	418641947	na
1093	chrM:15112	chrM	Assembly	418641948	na
1094	chrM:15295	chrM	Assembly	418641949	na
1095	chrM:15370	chrM	Assembly	418641950	na
1096	CH213-164F21:131490	H213-164F	Candidate	158145716	119103581
1097	CH213-164F21:169211	H213-164F	Candidate	158145772	119103637
1098	CH213-118G22:184516	H213-118G	Candidate	158145776	119103641
1099	CH213-119K16:14070	H213-119K	Evenly Distributed	418641977	na
1100	CH213-21C23:188808	H213-21C2	Evenly Distributed	418641953	na
1101	CH213-119K16:207645	H213-119K	Evenly Distributed	418641978	na
1102	CH213-21C23:67840	H213-21C2	Candidate	418641915	na
1103	chrUn:53966403	chrUn	Assembly	418641951	na
1104	chrUn:53966948	chrUn	Assembly	418641952	na
1105	chrUn:1279794	chrUn	Assembly	418642601	na
1106	chrUn:2154566	chrUn	Assembly	418642602	na
1107	chrUn:2474754	chrUn	Assembly	418642603	na
1108	chrUn:2632376	chrUn	Assembly	252841074	119104080
1109	chrUn:2776586	chrUn	Assembly	120258568	119103528
1110	chrUn:6149229	chrUn	Assembly	418642605	na
1111	chrUn:6720054	chrUn	Assembly	244222987	119103937
1112	chrUn:6889743	chrUn	Candidate	244222988	119103938
1113	chrUn:6940973	chrUn	Assembly	418642606	na
1114	chrUn:7283183	chrUn	Assembly	418642607	na
1115	chrUn:7381868	chrUn	Assembly	252841045	119104051
1116	chrUn:7866784	chrUn	Candidate	252841054	119104060

1117	chrUn:7934402	chrUn	Assembly	418642608	na
1118	chrUn:8025991	chrUn	Candidate	418642609	na
1119	chrUn:10213240	chrUn	Assembly	418642613	na
1120	chrUn:10540032	chrUn	Assembly	418642614	na
1121	chrUn:11085407	chrUn	Assembly	418642615	na
1122	chrUn:11980918	chrUn	Assembly	252841136	119104142
1123	chrUn:12390868	chrUn	Assembly	120258569	119103529
1124	chrUn:14043112	chrUn	Assembly	418642618	na
1125	chrUn:14127611	chrUn	Assembly	418642619	na
1126	chrUn:17470353	chrUn	Assembly	418642620	na
1127	chrUn:17560757	chrUn	Assembly	418642621	na
1128	chrUn:17704187	chrUn	Assembly	418642622	na
1129	chrUn:17922401	chrUn	Assembly	120258570	119103530
1130	chrUn:20217198	chrUn	Assembly	418642625	na
1131	chrUn:21213332	chrUn	Assembly	120258571	119103531
1132	chrUn:23042966	chrUn	Assembly	418642626	na
1133	chrUn:23384875	chrUn	Assembly	418642627	na
1134	chrUn:23753128	chrUn	Assembly	244222989	119103939
1135	chrUn:24511995	chrUn	Assembly	418642628	na
1136	chrUn:25691760	chrUn	Assembly	252841096	119104102
1137	chrUn:25831365	chrUn	Assembly	418642629	na
1138	chrUn:25946639	chrUn	Assembly	244222990	119103940
1139	chrUn:26026523	chrUn	Assembly	120258572	119103532
1140	chrUn:26305459	chrUn	Assembly	244222991	119103941
1141	chrUn:26389255	chrUn	Assembly	244222992	119103942
1142	chrUn:27040022	chrUn	Assembly	418642631	na
1143	chrUn:27149198	chrUn	Assembly	418642632	na
1144	chrUn:27402745	chrUn	Assembly	252841068	119104074
1145	chrUn:27478064	chrUn	Assembly	244222993	119103943
1146	chrUn:27589750	chrUn	Assembly	418642633	na
1147	chrUn:28158103	chrUn	Assembly	418642634	na
1148	chrUn:28314517	chrUn	Assembly	418642635	na
1149	chrUn:28671327	chrUn	Assembly	244222995	119103945
1150	chrUn:29017220	chrUn	Assembly	418642637	na
1151	chrUn:29087782	chrUn	Assembly	244222996	119103946
1152	chrUn:29831084	chrUn	Assembly	418642640	na
1153	chrUn:30223426	chrUn	Assembly	418642641	na
1154	chrUn:30545876	chrUn	Assembly	120258573	119103533
1155	chrUn:30606854	chrUn	Assembly	244222997	119103947
1156	chrUn:31339987	chrUn	Assembly	244222998	119103948
1157	chrUn:32210273	chrUn	Assembly	418642644	na
1158	chrUn:32307959	chrUn	Candidate	418642645	na
1159	chrUn:32523521	chrUn	Assembly	418642646	na
1160	chrUn:32765800	chrUn	Assembly	244222999	119103949
1161	chrUn:33627890	chrUn	Assembly	120258574	119103534
1162	chrUn:33873966	chrUn	Assembly	252841086	119104092
1163	chrUn:35285565	chrUn	Assembly	418642649	na
1164	chrUn:36334731	chrUn	Assembly	244223000	119103950
1165	chrUn:37016121	chrUn	Assembly	418642651	na
1166	chrUn:37631434	chrUn	Assembly	244223001	119103951
1167	chrUn:38378170	chrUn	Assembly	120258576	119103536
1168	chrUn:38561237	chrUn	Assembly	252841055	119104061
1169	chrUn:39760177	chrUn	Assembly	244223002	119103952
1170	chrUn:56537801	chrUn	Candidate	244223003	119103953
