# Supplementary material for: Phylogeography and adaptation genetics of stickleback from the Haida Gwaii archipelago revealed using genome-wide single nucleotide polymorphism genotyping
Source: Mol Ecol. 2013 Mar 4;22(7):1917–32. doi: 10.1111/mec.12215 (PMC3604130; doi:10.1111/mec.12215)
Supplement: Fig S2 — Neighbour-joining distance tree constructed as in Fig 2 but with poorly supported (50% bootstrap cut-off value) branches condensed to highlight the unresolved basal node and well-supported clusters at higher levels. [file mec0022-1917-sd2.pdf]

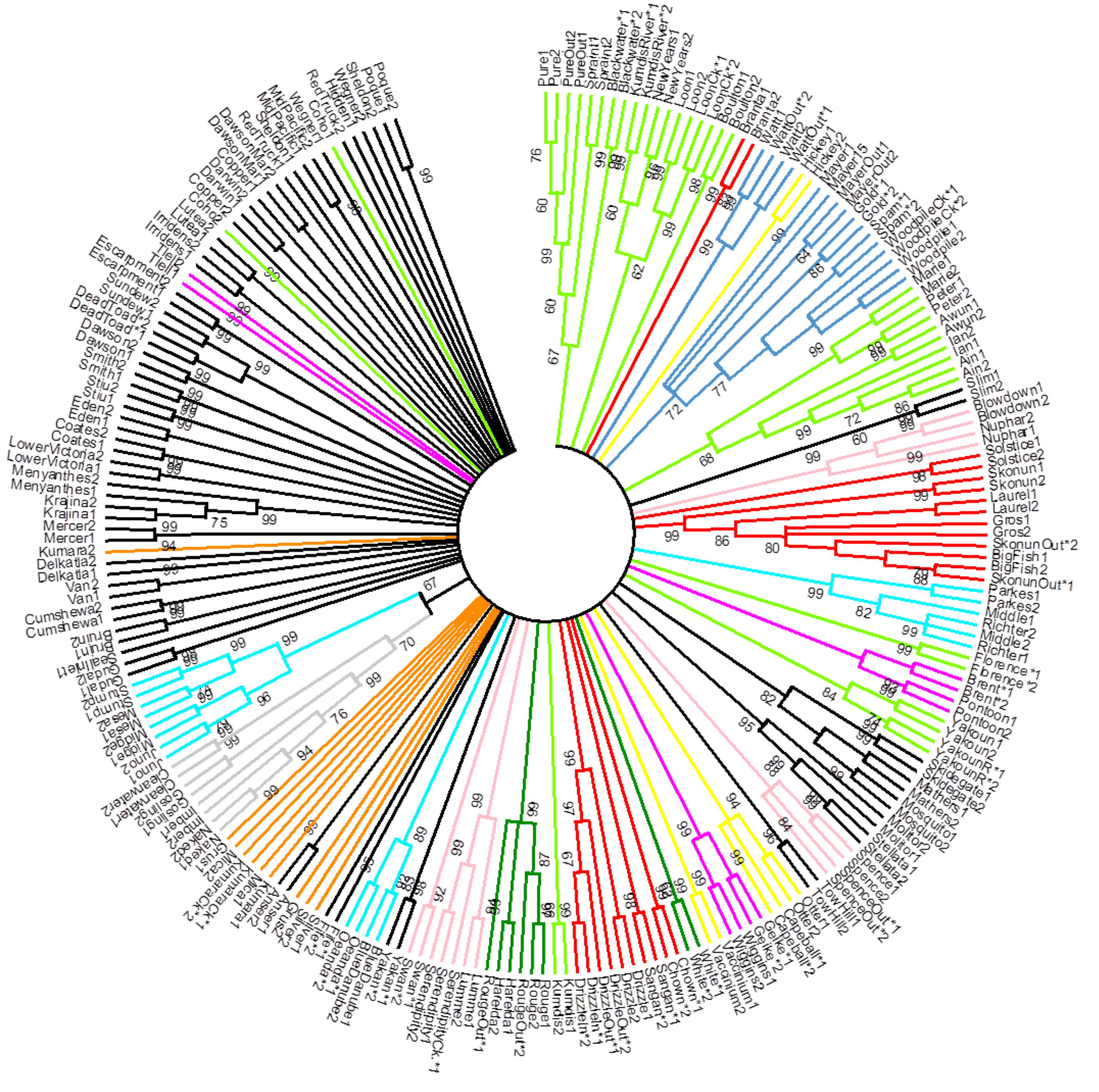

Fig. S2 Condensed neighbour-joining distance tree (50% cut-off value) constructed with SNP data from two stickleback from each of 115 Haida Gwaii localities (n= 227 individuals; 3 populations had only a single fish). Tree constructed as tree in Fig 2 and highlights the unresolved basal node and well-supported clusters at higher levels. Bootstrap values are shown next to branches.
